# Healthcare providers’ perceptions on barriers to the provision of psychosocial cancer care at Ocean Road Cancer Institute in Dar es Salaam, Tanzania

**DOI:** 10.1371/journal.pone.0361460

**Published:** 2026-10-28

**Authors:** Kibwanda Athumani, Nazima Dharsee, Masunga K. Iseselo, Rashidi Heri Kiangi, Dickson Ally Mkoka

**Affiliations:** 1 Department of Clinical Nursing, Muhimbili University of Health and Allied Sciences, Dar es Salaam, Tanzania; 2 Department of Clinical Oncology, Muhimbili University of Health and Allied Sciences, Dar es Salaam, Tanzania; 3 Department of Nursing Education and Management, Muhimbili University of Health and Allied Sciences, Dar es Salaam, Tanzania; The Hong Kong Polytechnic University, HONG KONG

## Abstract

**Background:**

Patients with cancer experience substantial psychosocial challenges throughout the cancer journey. Psychosocial cancer care is crucial for addressing patients’ psychological, emotional, social, spiritual needs, and for promoting well-being and quality of life. Despite its importance, psychosocial cancer care services remain insufficiently provided within routine cancer care services in many settings.

**Aim of the study:**

To explore healthcare providers’ perceptions of barriers to providing psychosocial cancer care services within routine care at a tertiary cancer hospital in Tanzania.

**Materials and methods:**

This study adopted an explorative qualitative design. This study was conducted at Ocean Road Cancer Institute in Dar es Salaam, Tanzania. Purposeful sampling was used to recruit 18 healthcare providers until information saturation was reached. Data were collected through face-to-face interviews using a semi-structured interview guide. All interviews were audio-recorded, transcribed verbatim, and analysed using a thematic analysis approach to identify key patterns and themes.

**Results:**

Three themes emerged that describe health care providers’ perceptions of barriers to the provision of psychosocial cancer care. These barriers include inadequately trained healthcare providers, an unsupportive organizational structure and environment for psychosocial care services, and challenges in establishing a trusting therapeutic relationship between patients and providers.

**Conclusion:**

Despite its recognized importance in promoting quality of life, the provision of psychosocial cancer care remains insufficiently provided in cancer settings. Findings from this study indicate that psychosocial care provision is limited by inadequate and incompetent staff, a lack of coordination, and an unsupportive organizational structure, and that building effective patient-provider therapeutic relationships was reported to hinder the provision of psychosocial care. To address these barriers, training, the establishment of coordinating units, and the development of protocols to guide the multidisciplinary team are essential. Integrating psychosocial care competencies into healthcare professionals’ training curriculum is also essential to bridge the gap.

## Introduction

Psychosocial struggles have been a common challenge among cancer patients and their families across the globe in various cancer settings. Despite these hardships, this aspect of care receives little attention [1]. It is estimated that between 16% and 65.9% of cancer patients in America, Australia, and Germany experience clinically significant psychosocial distress during the course of their illness [2]. In South Africa, more than 33% of cancer patients report substantial psychosocial distress [2]. Evidence indicates that patients with high disease burden, poor prognosis, and those aged 18–36 or over 65 years are at increased risk [2–5].

Psychosocial care is an essential component of comprehensive cancer care that addresses social, emotional, spiritual, and psychological concerns of patients and their families [6]. In cancer care settings, it extends beyond the management of physical symptoms to address psychosocial challenges associated with diagnosis, treatment, survivorship, and end-of-life care [7]. Providing psychosocial care throughout the cancer trajectory can help patients and families cope with emotional and social impacts of cancer and its treatment.

Evidence suggests that the effective provision of psychosocial care can enhance treatment adherence, improve survival, reduce treatment-related costs, and ultimately improve quality of life [6–9]. Accordingly, clinical practice guidelines and models, including those proposed by the Institute of Medicine (IOM), advocate for the integration of psychosocial care into routine cancer care [7]. Despite this evidence, psychosocial care remains underprioritized in routine cancer cares [6,7,10]. Therefore, it is essential to explore healthcare providers’ perceptions of psychosocial care provision comprehensively and within the contexts in which care is delivered.

Healthcare providers, especially oncology nurses, oncologists, and social workers, play a central role because they maintain more frequent contact with cancer patients. This position provides an opportunity to deliver psychosocial care alongside medical treatment. Health care providers’ competencies and understanding of psychosocial care are fundamental to its effective delivery. However, healthcare providers in oncology settings face significant challenges in providing high-quality psychosocial care [11,12]. These challenges include inadequate understanding of psychosocial care and a predominant focus on patients’ physical and medical needs [4,13]. Other reported barriers include limited time, inadequate training, competing professional responsibilities, communication barriers, and heavy workloads [8,10–12,14–16]. Despite their importance, these barriers remain insufficiently explored in the literature.

In Tanzania, efforts to improve cancer care have included the development of health strategic plans, training oncology nurse specialists, and the establishment of palliative care services [6,17–19]. Despite these efforts, the extent to which psychosocial care is provided to patients with cancer, families, and the community remains insufficiently addressed. Where provided, such care is often uncoordinated, fragmented, and inconsistent. The Provision of psychosocial care is inherently personal and subjective; therefore, the experience of frontline healthcare providers needs to be appraised within their distinctive socio-cultural contexts [12,20]. Exploring healthcare providers’ perceptions of psychosocial care and barriers they encounter in its provision is essential. This understanding can inform the development of appropriate psychosocial care strategies that are responsive to the local needs and aligned with the socio-cultural contexts.

## Materials and methods

### Study design

This study adopted an exploratory qualitative design. This design allowed the authors to gain a comprehensive understanding of perceived barriers to psychosocial care provision in the local context**.** It is particularly appropriate when there is limited information available in the existing literature on the issue under investigation [21].

### Study setting

This study was conducted at Ocean Road Cancer Institute (ORCI), a national tertiary cancer referral hospital in Dar es Salaam, Tanzania. The Institute provides comprehensive cancer services and receives patients referred from various regions in Tanzania and neighbouring countries, including Zambia, Malawi, Comoros, and Mozambique. Currently, the ORCI receives more than 65,000 patient visits annually, including approximately10,000 new patients and 55,000 follow-up visits. Its core services include cancer treatment, early detection, prevention, and teaching.

ORCI has ten directorates. The medical and allied health sciences services directorate deals with screening, early detection, diagnosis, and treatment. The directorate comprises ten sections, including clinical oncology, surgical oncology, pharmacy, Nursing, Laboratory, Radiotherapy and imaging, medical physics, Radiotherapy technology, and palliative care. At the time of the study, the social workers were administratively located within the nursing section.

ORCI did not have a specialized unit dedicated specifically to psychosocial cancer care. Instead, it was primarily provided through a palliative care team, comprising palliative care nurses, pharmacists, oncologists, and social workers. Oncologists and pharmacists were unavailable during the data collection period due to other equally important institutional commitments; hence, they were excluded from the study. Although social workers were available to address psychosocial concerns, their administrative placement within the nursing section and absence of a dedicated psychosocial care unit may have contributed to unclear reporting and referral pathways. These organizational arrangements were perceived to contribute to fragmented delivery of psychosocial care. The palliative care team served as an important source of psychosocial and supportive care for patients with cancer. However, participants reported that services were predominantly focused on patients with advanced or end-stage disease, leaving many patients at earlier stages of their cancer journey with limited structured psychosocial support.

### Recruitment of study participants

Participants were selected using purposive sampling to recruit individuals able to provide rich information relevant to the study questions. We recruited 18 participants: 15 nurses and three social workers. The first two authors (KA and ND), who had experience working with cancer patients, recruited participants based on predetermined inclusion criteria. Eligible participants were healthcare providers working at ORCI in the current unit for at least six months, who were directly involved in the provision of care to patients with cancer, willing to provide informed consent, and able to communicate either in Kiswahili or English. Participants were selected to achieve variations in durations of professional experience, work units, sex, educational backgrounds, age, and professional cadre. Potential participants were approached by the researcher and provided with information about the study before deciding whether to participate.

### Data collection methods and procedures

Participants’ recruitment and interviews took place between 20^th^ April and 19^th^ May 2022. Potential participants were approached by the researcher in collaboration with the relevant unit in charge and informed about the purpose and procedures of the study. For Participants who agreed to participate, the interview appointments were scheduled approximately two days in advance by the first author (KA), an oncology nurse specialist with training and experience in qualitative research.

Face-to-face individual interviews were conducted at participants’ respective working units at mutually agreed times. Interviews were conducted after participants’ working hours to minimize interruptions to clinical services. The first author (KA) conducted all the interviews using a semi-structured interview guide with open-ended questions regarding barriers to providing psychosocial care for cancer patients attending ORCI. The guide consisted of four broad questions: (1) What do you understand by the term psychosocial care in cancer patients? (2) What are the challenges or barriers you often face during the provision of psychosocial care to cancer patients? (3) How does the hospital organization structure or environment affect the provision of psychosocial care? (4) What challenges do you experience when trying to establish a therapeutic relationship with patients?

Main questions were followed by specific probes to encourage participants to elaborate on their experiences and perceptions. The guide was developed following a review of relevant literature. KA and ND, who had expertise in oncology, contributed to the development, while other co-authors provided input regarding qualitative research principles and relevance of the questions to the study objectives. The guide was refined during data collection in response to emerging issues and areas requiring further probing

Before each interview, participants were informed about the study objectives, the voluntary nature of participation, and their right to decline participation or withdraw without consequences. Written and oral informed consents were obtained before the interview. Separate consent was obtained for audio recording. Participants were informed that their identities would not be disclosed and that numerical codes would be used instead of names. All interviews were conducted in Kiswahili, the language commonly spoken by participants. Data collection continued until the 18^th^ interview, when the researchers judged that further interviews were yielding largely repetitive information with little additional insight relevant to research questions**.** Each interview lasted between 30 and 48 minutes.

### Data quality and management

Several measures were taken to ensure data quality and accuracy. The first author (KA) transcribed each audio-recorded interview verbatim, on the same day or shortly after data collection. Each Transcript was compared with its corresponding audio recording to verify accuracy. Collected data and transcripts were also periodically shared with the second and last authors (DAM and ND) for quality review. Feedback from these reviews informed refinement of probing questions and interview techniques during subsequent interviews.

Interviews were conducted in Kiswahili. The translation into English was undertaken during the coding and theme development process to preserve the meaning of participants’ accounts while facilitating collaborative analysis. Participants’ identifiers were removed from each transcript and replaced with numerical codes. All data were securely stored in three different password-protected devices. Electronic research files were stored on password-protected devices, and files shared among members of the research team were encrypted and password-protected. Access to the data was restricted to authorized members of the research team. Research folders, transcripts, coding materials, and the codebook were systematically labelled and maintained to facilitate secure data management and retrieval.

### Data analysis process

Data were analysed using a thematic analysis informed by the approach described by Braun and Clarke [22]. Each transcript was read multiple times to enhance familiarity with the data. The analysis was guided by research questions, particularly participants’ perceptions of barriers to the provision of psychosocial care and psychosocial care needs of patients with cancer as predetermined thematic areas. Relevant sections of the transcripts were manually coded by KA, using Microsoft Excel. Initial codes were compared and discussed with co-authors (ND, MKI, RHK, and DAM) through peer debriefing. Codes were then reviewed, refined, and grouped into sub-themes. These were then clustered into broader themes that captured recurring patterns and underlying meanings within participants’ accounts. Although the research questions provided initial areas of focus, the analysis remained responsive to the issues that emerged from participants’ narratives.

Field notes were also reviewed during interpretation to provide contextual information and support clarification of emerging concepts. The research team held discussions at different stages of analysis to examine the coherence of codes, sub-themes, and themes. The final findings were supported with representative participants’ quotations, identified using numerical codes to protect confidentiality.

### Reflexivity

The principal investigator (KA) was an oncology nursing student who had practised at ORCI for more than a year before data collection. This prior experience provided an in-depth understanding of the clinical context and challenges surrounding psychosocial care, but also had the potential to influence data collection and interpretation of the findings.

To minimize the influence of prior experience and assumptions, KA maintained a reflexive memo throughout data collection and analysis. The notes documented feelings, experiences, personal assumptions, and reactions related to the research topic. Peer debriefing was also undertaken throughout the research process. The interview guide, transcripts, codes, subthemes, and themes were shared with co-researchers who were not directly involved in the interviews and without an oncology background (DAM, MI, and RH). These discussions enabled the research team to critically examine interpretations and ensure that the findings remained grounded in the participants’ perspectives.

### Trustworthiness

To ensure that findings are worth believing, four criteria were applied, including credibility, transferability, dependability, and conformability [23]. The credibility of the study was enhanced by recruiting participants from different professional and clinical backgrounds, including nurses and social workers, which enabled triangulation of results. Also, Prolonged engagement in the study setting through the first author’s clinical experience facilitated rapport and contextual understanding. Repeated review of transcripts, peer debriefing, and collaborative discussion of codes, subthemes, and themes enhanced the credibility of this study.

Transferability was supported by thick description of the study setting, participants, recruitment procedures, data collection procedures, and analysis process. This allows readers to assess the relevance of the findings in other cancer settings. Dependability was supported by clear and proper documentation of research procedures, including recruitment, interview process, coding, theme development, and all changes made to the interview guide during data collection. Confirmability was ensured through reflexive memo writing, peer debriefing, collaborative analysis process, and the use of direct quotations.

### Ethical consideration

Ethical approval of this study was obtained from the Institutional Review Board of Muhimbili Health and Allied Sciences (MUHAS), Tanzania (IRB No. MUHAS-REC-03-2022-1066). Permission to conduct the study was also obtained from the management of ORCI. Written and oral informed consent were obtained from all participants before the interviews. Participants were informed about the purpose of the study, the voluntary nature of participation, and the right to decline or withdraw from the study without negative consequences. They were also informed about the use of audio recording before the interview. To maintain confidentiality, participants were assigned numerical identification codes, which were used during interviews, transcriptions, analysis, and reporting instead of names or other direct identifiers. Audio recordings and transcripts were stored on password-protected devices. Also, all electronic files shared with other members of the research team were encrypted and password-protected.

## Findings

### Participants’ socio-demographic characteristics

A total of 18 healthcare providers participated in the study, consisting of 15 registered nurses and three social workers. Participants had a mean age of 44.47 ± 10.86 years**,** with their ages ranging from 28 to 60 years. Thirteen participants were females, and five were males. Their experience caring for cancer patients ranged from 3 to 34 years. Six participants held a bachelor’s degree in nursing or social work, seven had a diploma in nursing, three had a master’s in nursing, and the rest had a certificate in nursing. None of the participants reported having attended a formal course specifically focused on psychosocial care provision. Table 1 presents the distribution of socio- demographic and professional characteristics of study participants.

**Table 1:** Socio-demographic and professional characteristics of study participants (n = 18).

Variable	Category	Frequency(n)	Percentage (%)
Age (in Years) Mean± SD(Range)		**18**	44.47 **± 10.86(**28-60)
Age categories	25-35	6	33.3
	36-55	6	33.3
	56-60	6	33.3
Gender	Female	13	72.2
	Male	5	27.8
The highest level of professional Education attained	Certificate	2	11.1
	Diploma	7	38.8
	Bachelor’s degree	6	33.3
	Master’s degree	3	16.6
working Unit	Chemotherapy	2	11.11
	OPD	1	5.56
	In patient	6	33.33
	Radiotherapy	1	5.56
	Palliative care and CTC	4	22.22
	Screening	1	5.57
	Social welfare	3	16.67
Attended any course for psychosocial care provision	No	18	100
	yes	0	0
Total number of years of employment in professional(years)±SD(Range)	16.00 ± **9.86(3-34)**		
Duration of working with cancer patients(years) ±SD(Range)	15 ± **9.73(3-34)**		

### Themes and subthemes emerged under barriers to psychosocial care provision

From the analysis of the interviews, three themes emerged that described health workers’ perceptions of barriers to the provision of psychosocial care to patients attending cancer services. These included inadequately trained staff for providing psychosocial care, an unsupportive organizational structure and environment, and challenges in establishing a trusting relationship between providers and patients. Table 2 presents the themes, subthemes, and representative codes.

**Table 2:** Themes and subthemes related to health workers’ perceptions of barriers to providing psychosocial care to patients attending cancer services.

S/No	Themes	Subthemes	Examples of codes
1.	Inadequately trained healthcare providers for providing psychosocial care	• Inadequate number of trained staff • Overwhelmed with excessive workload	• I feel incompetent to provide it • Don’t have enough time to provide PSC • high workload
2.	Unsupportive organizational structure and environment for psychosocial care services	• Inadequate supportive resources • Improper organization and coordination	• There is no chaplain’s office at our setting • No hostels for caregivers • There are no guidelines for provision of psychosocial care
3.	Challenges in establishing a patient-provider trusting relationship	• Inadequate skills for therapeutic communication • Patients’ hesitancy in sharing problems	• I don’t know how to talk about it • Patients don’t like to expose their psychological problems • Patients don’t trust nurses

### Theme 1. Inadequately trained healthcare providers for providing psychosocial care

#### (a) Inadequate number of trained staff.

Participants in this study reported that the number of hospital providers was insufficient compared to the growing demand for oncological care. In addition, several participants perceived a gap in psychosocial care competencies among health workers caring for cancer patients. They noted that they had not received formal training in psychosocial care. They also acknowledged that most of their on-the-job training focused primarily on the biomedical dimensions of patients’ diseases, with little or no emphasis on patients’ psychosocial needs. As a result, participants felt inadequately prepared to deliver comprehensive psychosocial care due to a lack of appropriate knowledge and skills. This can be attested by one of the participants below.

*“I have not received training on psychosocial care; most of the training I have been receiving is on pain management. Those related to psychosocial care… no!! So, I feel incompetent to attempt such a big thing” [Participant 1; Nurse].*

#### (b) Overwhelmed with excessive workload.

Furthermore, participants acknowledged that addressing the psychosocial needs of patients with cancer required providers to dedicate additional time. However, interviews reported that health workers were few and burdened with multiple responsibilities. It was noted that there were no trained staff to run services such as psychosocial care. Interviewees were concerned that excessive workload and other equally important caring tasks limited the available time to explore the psychosocial needs of their patients. One participant narrated this below.

*“We do try our level best to explore patient needs, as you know, the burden is huge, because patients are many and staff is not enough, so you find at some point I have to be in ward rounds or dispensing medications, so the time to talk with patients in detail is minimal.” [Participant 2; Nurse].*

### Theme 2. Unsupportive organizational structure and environment for psychosocial care services

#### (a) Improper organization and coordination.

Participants perceived psychosocial care as poorly structured, coordinated, and supported within the hospital setting. Several participants expressed concerns about the lack of structured and organised services that address the psychosocial needs of patients and their relatives. Participants believed that psychosocial care was less prioritised by management because the hospital lacked a dedicated unit or focal point to oversee the service. Although Social workers were available, participants reported that their organizational placement within the nursing section and a lack of clearly defined roles and coordination mechanisms made collaboration with other professionals less systematic. Participants thought that this arrangement could result in unclear referral pathways, delayed involvement of appropriate professionals, and fragmentation of psychosocial care. Furthermore, Participants also described difficulties in coordinating spiritual care. Spiritual leaders were considered to be important in addressing psychosocial needs of patients; however, their involvement within routine clinical services was reported to be less coordinated.

*“You may call a priest for providing spiritual care, but when they come, they are not given time to attend patients, or they have to wait for a very long time to allow the clinician to finish attending to the patients,”* [Participant 9, Nurse]

Establishment of clear working relationships and links also emerged, in which participants commented on the need for proper coordination between biomedical and psychosocial services to minimize unnecessary inconvenience in delivery of care; Furthermore, they insisted on teams of health care professionals who are involved in provision of psychosocial care to work cohesively not only at the time when the disease has reached advanced stage but also to the other stages of the illness.

*“Sometimes spiritual services overlap with other medical services, leading other nurses to delay or prohibit them. Therefore, all staff are supposed to be informed and guided on how to manage these services to remove inconvenience; you know the issue of faith has a greater impact than even the treatment we provide” [Participant 16, Social worker].*

#### (b) Inadequate supportive resources.

As it was noted during interviews, the hospital has no office for chaplain services. Also noted that the size of the church and mosque is very small and that the spiritual leaders (pastors or sheikhs) were not fully time-committed, making spiritual care less accessible and not delivered on time when needed.

*Sometimes it’s difficult to reach spiritual leaders by phone, probably because they’re busy with other commitments. Here, we don’t have offices where they could stay and deliver spiritual services on time. Additionally, the church or mosque is small* [Participant 3, Nurse].

A few participants reported attempting to meet their patients’ psychosocial needs. However, they were unsure whether what they had been doing was correct because there were no guidelines or protocols to guide them.

*‘Here, in fact, there are no guidelines for diagnosing and providing psychosocial care. I probably have not seen it, and I personally use experience”* [Participant 8; Nurse].

In addition, participants emphasized the importance of creating a motivating work environment, recognizing individual contributions, and increasing wages as approaches to empower staff in providing psychosocial care.

*“I think administration should consider putting in place user-friendly working aids, recognizing each staff effort and raising their wages for the extra time they devote to deliver the care” [Participant 18, Social worker]*

### Theme 3. Challenges in establishing a patient-provider trusting relationship

#### (a) Inadequate skills for therapeutic communication.

Participants described some difficulties they encountered in building rapport with their patients. Limited time and inadequate communication skills were identified as major barriers to establishing good relationships between patients and health care providers. While Participants acknowledged that effective communication skills are a crucial pillar for establishing a therapeutic relationship, they often felt unprepared to elicit patients’ psychosocial concerns or to initiate conversations that might reveal such needs. Some expressed fear of not being able to respond appropriately to patients’ psychological needs or provide the right information when addressing such concerns. One participant said.

*“I personally don’t get too much involved in asking patients about psychosocial things for fear of not being able to find good words to tell them. Many of us do the same as I do”* [Participant 11, Nurse].

#### (b) Patients’ hesitancy in sharing problems.

Additionally, participants expressed concerns about patients’ hesitancy to share their psychosocial needs or concerns even when health workers made an effort to probe them during assessment. It was also noted that this hesitancy stemmed from patients’ perceptions that health care providers were untrustworthy and unable to offer meaningful help for their concerns.

*“You might counsel someone, but he or she can ask you, ‘Are you God?” You can hear them say, “You know, doctors are liars. They can tell you it’s ok, but in reality, they know you are dying”. As a result, the patient feels that we, health workers, tend to hide someone’s illness. Hence, they lose trust in us, and because of that, they don’t open up to share their issues.” [Participant 6, Nurse].*

However, some patients were willing to share their concerns when engaged in more in-depth discussion with health workers, as reported by one participant below.

*“For patients to disclose their issues or concerns, it has been a big problem here, until you dig deep during your conversation with them” [*Participant 17, Social *worker]*

Personal distress arising from daily care for patients with cancer, along with workplace challenges, was reported to interfere with health workers’ ability to demonstrate empathy, compassion, and thoughtful consideration to patient concerns. One participant attests to this

*“Sometimes I might have a desire to convey love, empathy, and listen to patients’ concerns or issues, but because of stresses emanating from the challenging work of caring for cancer patients, I find myself unable to do so” [Participant 4, Nurse].*

## Discussion

This study explored healthcare providers’ perceptions of barriers to psychosocial care services within routine cancer care. Three interconnected barriers emerged: inadequate number of trained staff, an unsupportive organizational structure and environment, and challenges in establishing trusting patient-provider therapeutic relationships. These findings indicate that barriers to care operate at multiple levels, ranging from individual provider competencies and workload to organizational systems and patient-provider interactions.

This study found that staff shortages, high patient volume, and competing clinical responsibilities constrained psychosocial care in the oncology unit. Cancer patients experience diverse psychosocial concerns, including emotional distress, limited support, and Financial stress related to high treatment costs [24]. Hence, providing psychosocial support requires healthcare providers to spend time listening to patients, exploring concerns, providing information, and facilitating appropriate referrals. However, participants described the situation in which routine clinical responsibilities often took priority over psychosocial discussions. This suggests that high workload and competing clinical responsibilities may limit healthcare providers’ ability to address psychosocial needs, particularly where staffing is inadequate. Comparable findings have been reported in Singapore and Turkey, where over-emphasis on biomedical needs and lengthy treatment processes constrained psychosocial care delivery [12,20]. Similarly, Abigiya et al. [25] identified staff shortages as a limiting factor to effective provision of psychosocial care in cancer units. These findings highlight that psychosocial care should not be viewed as an additional activity that can be taken only when time permits. Rather, it needs to be integrated into routine cancer care processes and supported through adequate staffing, appropriate referral mechanisms, and organizational prioritization.

Our study revealed that healthcare providers were not adequately prepared to provide psychosocial care. Participants reported that they had not received formal training on psychosocial care and felt inadequately prepared to identify and respond to patients’psychosocial concerns. This finding concurs with previous studies which show that insufficient knowledge, skills and confidence among oncology healthcare providers can limit the integration of psychosocial care into routine practice [16,26]. These findings suggest that oncology healthcare providers require communication, assessment, supportive counselling, referral, and boundary-setting skills to respond safely and effectively to patient needs. Therefore, incorporating psychosocial care into oncology education and providing on-the-job training may strengthen providers’ confidence and capacity to address patients’ psychosocial concerns.

An unsupportive organizational structure and environment were identified as important barriers to psychosocial care provision. Provision of psychosocial care requires effective organization and a supportive environment within oncology care hospitals [27]. Participants described the absence of a dedicated psychosocial care unit, inadequate supportive resources, unclear coordination, and lack of guidelines or protocols. Although social workers and spiritual care providers were available, their involvement was perceived as insufficiently integrated into routine multidisciplinary cancer care. This finding aligns with evidence emphasizing the importance of organizational structure and a multidisciplinary approach in providing comprehensive psychosocial care in oncology services [27–30]. Without clear referral pathways, defined professional roles, and effective communication, psychosocial care may remain fragmented and depend on individual provider initiatives.

The organizational position of social workers is particularly relevant in this context. Participants reported that social workers were administratively located within the nursing section and that their roles in psychosocial care were not always clearly coordinated with those of other professionals. Such organizational arrangement may create uncertainty regarding responsibility for psychosocial assessment, referral, follow-up, and coordination, potentially leading to fragmented delivery of psychosocial care. Previous studies emphasize the importance of a clearly organized multidisciplinary structure, defined professional roles, and proper coordination in delivery of psychosocial care [27,31]. Similarly, Organizational support and clearly established systems have been identified as important factors in integrating psychosocial care into routine cancer services [30]. This finding does not imply that placement within the nursing section is inherently inappropriate. Rather, it just highlights the significance of clearly defining responsibilities, communication channels, and multidisciplinary referral pathways regardless of organizational structure. Therefore, establishing coordinated care with clearly defined roles and a referral system may improve accessibility, continuity, and integration of psychosocial care within routine oncology services.

This study also identified inadequate supportive resources, including limited space for spiritual care and the absence of clear guidelines and protocols for psychosocial oncology care. Participants reported that spiritual leaders were not readily accessible and that available facilities were inadequate. The absence of standardized guidelines may further contribute to variations in practice because healthcare providers may rely on personal experience. Hence, developing contextually relevant psychosocial protocols and guidelines could support delivery of standardized psychosocial care.

The challenges in establishing a trusting relationship between healthcare providers and patients also emerged as a major barrier in this study. The participants reported difficulties in initiating conversations about psychosocial care concerns, limited communication skills, insufficient time, patients’ hesitancy to disclose information, and mistrust of healthcare providers. These findings are consistent with previous research indicating that health care providers may feel uncertain about how to initiate psychosocial discussions and respond to emotional concerns [14,20]. The finding that some healthcare providers avoided asking about psychosocial concerns because they were unsure what to say is particularly important. This implies that communication skills training should go beyond to include how to initiate difficult conversations, responding to emotional distress, maintaining therapeutic boundaries, and making appropriate referrals.

Participants also perceived mistrust and hesitancy to disclose psychosocial concerns as barriers to care. They described that some patients believed healthcare providers might withhold information about prognosis or provide reassurance that was inconsistent with the patient’s understanding about their illness. Such perceptions may reduce patients’ willingness to disclose emotional and social concerns. Building trust therefore requires not only communication skills but also consistency, transparency, respect, privacy, and adequate time for meaningful interaction. Previous studies similarly emphasize the importance of patient-provider communication in patient psychosocial well-being and experience in cancer care [32–34].

An important finding from this study that requires special attention was the effect of healthcare providers’ own emotional and occupational stress on their ability to provide psychosocial care. They described stresses arising from the challenges of caring for cancer patients and other non-clinical responsibilities that reduced their capacity to engage empathetically with patients. This finding indicates how occupational stresses and emotional exhaustion may affect providers’ capacity to engage empathetically with patients. This highlights the gap that exists between providers’ intention to provide psychosocial care and emotional capacity to do so. This account is similar to literature showing that healthcare providers caring for patients with cancer experience considerable emotional exhaustion and burnout [32,35,36]. This may in turn interfere with providers’ attentiveness, empathy, communication, and ability to maintain therapeutic interaction. Therefore, interventions aimed at strengthening psychosocial care should address not only patient needs but also emotional demands and occupational demands placed on healthcare providers [35,36].

## Conclusion

Psychosocial cancer care is a critical component for oncology services that address the psychological, social, and spiritual needs of patients with cancer and their families. Effective provision of psychosocial cancer care in routine cancer care improves the well-being and quality of life of patients and their families. This study found that the provision of psychosocial care is hindered by a shortage of trained staff working in settings with competing care demands, the absence of a structured, coordinated, and supportive environment for psychosocial delivery, and challenges in establishing a trusting therapeutic relationship between healthcare providers and patients. Therefore, establishing well-organised and coordinated psychosocial care systems, and the integration of psychosocial care into pre-service and in-service training curricula may enhance provision psychosocial care services.

## Implication of the study

These findings highlight the need for the hospital management to design dedicated units or a coordination system within the hospital structure to strengthen the organization and delivery of psychosocial cancer care services. In addition, healthcare providers should receive formal training to enhance their competencies in psychosocial care. Also, development of clear protocols and collaborative frameworks is needed to support the integration of psychosocial care into routine cancer care.

Training institutions should incorporate psychosocial cancer care competencies in their curricula to address the existing knowledge and skills gap of future healthcare providers. Also, future research should include patients, hospital managers, and professional groups not represented in this study to obtain a broader understanding of barriers and facilitators to psychosocial cancer care.

## Study limitations

This study had several limitations. First, the study was conducted at a single national cancer referral; therefore, the findings may not fully represent the experience in other cancer settings in Tanzania. Second, some important stakeholders, including other professional cadres, patients, and hospital management, were not interviewed. Their perspectives could have provided additional insights into organizational and systemic barriers affecting psychosocial care. Therefore, future studies should include these populations for a more holistic understanding. Third, recruiting healthcare workers for an interview was challenging due to their demanding schedules and limited numbers, which compromised participation. Although individual interviews provided an opportunity for participants to describe their experience, the use of participant groups in future studies could provide triangulation of the findings.

## References

[1] Jacobsen PB, Lee M. Integrating psychosocial care into routine cancer care. Cancer Control. 2015;22(4):442–9. doi: 10.1177/107327481502200410 26678971

[2] Mehnert A, Hartung TJ, Friedrich M, Vehling S, Brähler E, Härter M, et al. One in two cancer patients is significantly distressed: Prevalence and indicators of distress. Psychooncology. 2018;27(1):75–82. doi: 10.1002/pon.4464 28568377

[3] Bultz BD, Groff SL, Fitch M, Blais MC, Howes J, Levy K, et al. Implementing screening for distress, the 6th vital sign: A Canadian strategy for changing practice. Psychooncology. 2011;20(5):463–9. doi: 10.1002/pon.1932 21456060

[4] Bultz BD, Carlson LE. Emotional distress: The sixth vital sign--future directions in cancer care. Psychooncology. 2006;15(2):93–5. doi: 10.1002/pon.1022 16444764

[5] Geue K, Brähler E, Faller H, Härter M, Schulz H, Weis J, et al. Prevalence of mental disorders and psychosocial distress in German adolescent and young adult cancer patients (AYA). Psychooncology. 2018;27(7):1802–9. doi: 10.1002/pon.4730 29644783

[6] United THE, Of R, Health MOF, Development C. National guidelines for the provision of psychosocial care and support services, vol. 1. Dar Es Salaam; 2022.

[7] Turnbull Macdonald GC, Baldassarre F, Brown P, Hatton-Bauer J, Li M, Green E, et al. Psychosocial care for cancer: A framework to guide practice, and actionable recommendations for Ontario. Curr Oncol. 2012;19(4):209–16. doi: 10.3747/co.19.981 22876147 PMC3410830

[8] Fann JR, Ell K, Sharpe M. Integrating psychosocial care into cancer services. J Clin Oncol. 2021;30(11):1178–86. 10.1200/JCO.2011.39.7398 22412139

[9] Care P, Holland JC, York N, Steensma DP. Caring for the whole patient: The science of. Am Soc Clin Oncol. 2012;30:29–59. 10.1200/JCO.2011.41.4078 22412125

[10] Abrahamson K, Durham M, Norton K, Doebbeling BN, Doebbeling CC, Anderson JG. Provision of psychosocial care for cancer patients: Service delivery in urban and rural settings. J Prim Care Community Health. 2011;2(4):220–4. doi: 10.1177/2150131911407201 23804837

[11] Chambers SK, Grassi L, Hyde MK, Holland J, Dunn J. Integrating psychosocial care into neuro-oncology: challenges and strategies. Front Oncol. 2015;5:41. doi: 10.3389/fonc.2015.00041 25756038 PMC4337368

[12] Chen CS, Chan SW-C, Chan MF, Yap SF, Wang W, Kowitlawakul Y. Nurses’ perceptions of psychosocial care and barriers to its provision: A qualitative study. J Nurs Res. 2017;25(6):411–8. doi: 10.1097/JNR.0000000000000185 29099474

[13] Patel MI, Lopez AM, Blackstock W, Reeder-Hayes K, Moushey EA, Phillips J, et al. Cancer disparities and health equity: A policy statement from the American Society of Clinical Oncology. J Clin Oncol. 2020;38(29):3439–48. doi: 10.1200/JCO.20.00642 32783672 PMC7527158

[14] Dilworth S, Higgins I, Parker V, Kelly B, Turner J. Patient and health professional’s perceived barriers to the delivery of psychosocial care to adults with cancer: A systematic review. Psychooncology. 2014;23(6):601–12. doi: 10.1002/pon.3474 24519814

[15] Weis J. Psychosocial care for cancer patients. Breast Care. 2015;10(2):84–6. 10.1159/000381969 PMC4463789 26195935

[16] Uwayezu MG, Nikuze B, Maree JE, Buswell L. Competencies for nurses regarding psychosocial care of patients with cancer in Africa: An imperative for action special articles abstract. 2022:1–12. 10.1200/GO.21.00240 PMC8789211 35044834

[17] Luyirika E, Med MF, Lohman D, Ali Z, Atieno M, Mahenge A, et al. Progress update: Palliative care development between 2017 and 2020 in five African countries. J Pain Symptom Manage. 2022;63(5):729–36. doi: 10.1016/j.jpainsymman.2021.12.026 34973351

[18] Ramaiya MK, Sullivan KA, Donnell KO, Cunningham K, Shayo AM, Mmbaga BT, et al. A qualitative exploration of the mental health and psychosocial contexts of HIV-positive adolescents in Tanzania. 2016:1–13. 10.1371/journal.pone.0165936 PMC5112865 27851797

[19] Oyoe N, Oti O, Villiers MD. Strengthening of oncology nursing education and training in Africa in the year of the nurse and midwife: Addressing the challenges to improve cancer control in Africa. 1–15. 10.3332/ecancer.2021.1209 PMC8057781 33912234

[20] Güner P, Hiçdurmaz D, Kocaman Yıldırım N, İnci F. Psychosocial care from the perspective of nurses working in oncology: A qualitative study. Eur J Oncol Nurs. 2018;34:68–75. doi: 10.1016/j.ejon.2018.03.005 29784141

[21] Hunter DJ, Howes D. Defining exploratory-descriptive qualitative (EDQ) research and considering its application to healthcare. J Nurs Heal care. 2019;4(1).

[22] Maguire M, Delahunt B. Doing a thematic analysis: A practical, step-by-step guide for learning and teaching scholars. 2017;3(3).

[23] Editor D. J Med Surg Public Health. 2024;2:0–3.

[24] Mensah NA, Mensah YB, Dedey F. Navigating the challenging storms of cancer management in a national cancer centre: Perspectives of female patients. BMC Public Health. 2024;24(1):2856. doi: 10.1186/s12889-024-20360-9 39415160 PMC11484427

[25] Wondimagegnehu A, Abebe W, Hirpa S, Kantelhardt EJ, Addissie A, Zebrack B, et al. Availability and utilization of psychosocial services for breast cancer patients in Addis Ababa, Ethiopia: A mixed method study. 2023;2023.

[26] Bergerot C, Jacobsen PB, Rosa WE, Lam WWT, Dunn J, Fernández-González L, et al. Global unmet psychosocial needs in cancer care: Health policy. EClinicalMedicine. 2024;78:102942. doi: 10.1016/j.eclinm.2024.102942 39634034 PMC11615525

[27] Schuit AS, Holtmaat K, van Zwieten V, Aukema EJ, Gransier L, Cuijpers P, et al. Organizing psycho-oncological care for cancer patients: The patient’s perspective. Front Psychol. 2021;12:625117. doi: 10.3389/fpsyg.2021.625117 33967892 PMC8100060

[28] Hospital and community-based psychosocial care for patients with cancer and their families: A model of care for psycho-oncology. 2020.

[29] Aji LM, Muhammad AB. Psychosocial care. 1–27.

[30] Recklitis CJ, Syrjala KL, Hutchinson F. HHS public access. 2018;18(1):1–23.

[31] Palos GR, Tortorella FR, Stepen K, Rodriguez MA. A multidisciplinary team approach to improving. 2013;17(5):2013. 10.1188/13.CJON.556-558 24080057

[32] Kriska R, Karina MP, Gergen-barnett K, Brimhall K, Ko NY. A review of medical mistrust across the cancer continuum of care and current interventions. 2025:750–60. 10.1007/s10900-025-01462-w PMC12301279 40123044

[33] Li C, Matthews AK, Dossaji M, Fullam F, Matthews AK, Dossaji M, et al. The relationship of patient – provider communication on quality of life among African-American and white cancer survivors. J Health Commun [Internet]. 2017;22(7):584–92. Available from: doi: 10.1080/10810730.2017.1324540 PMC6077992 28581896

[34] Hong YR, Yadav S, Erim D, Turner K. Patient – provider discussion about emotional and social needs, mental health outcomes, and benefit finding among U. S. adults living with cancer. 2021:3622–34. 10.1002/cam4.3918 PMC8178502 33960716

[35] Lyu X-C, Jiang H-J, Lee L-H, Yang C-I, Sun X-Y. Oncology nurses’ experiences of providing emotional support for cancer patients: A qualitative study. BMC Nurs. 2024;23(1):58. doi: 10.1186/s12912-024-01718-1 38245735 PMC10800062

[36] Civan Kahve A, Çapar DG, Keleş MK, Çınar MA, Uçar G, Perkin P, et al. Psychological effects of being a healthcare worker and a caregiver for cancer patients: A comparative analysis. Support Care Cancer. 2025;33(7):633. doi: 10.1007/s00520-025-09699-w 40571860 PMC12202510

