# Supplementary material for: Healthcare providers’ perceptions on barriers to the provision of psychosocial cancer care at Ocean Road Cancer Institute in Dar es Salaam, Tanzania
Source: PLoS One. 2026 Oct 28;21(10):e0361460. doi: 10.1371/journal.pone.0361460 (PMC13716222; doi:10.1371/journal.pone.0361460)
Supplement: Supplementary file 7 [file pone.0361460.s007.docx]

**Response to Academic Editor comments**

**Comment:** **1**	If the reviewer comments include a recommendation to cite specific previously published works, please review and evaluate these publications to determine whether they are relevant and should be cited. There is no requirement to cite these works unless the editor has indicated otherwise

**Response:** No recommendation was raised by reviewers who requested to cite previously published work

**Comment 2.** Please review your reference list to ensure that it is complete and correct. If you have cited papers that have been retracted, please include the rationale for doing so in the manuscript text, or remove these references and replace them with relevant current references. Any changes to the reference list should be mentioned in the rebuttal letter that accompanies your revised manuscript. If you need to cite a retracted article, indicate the article’s retracted status in the References list and also include a citation and full reference for the retraction notice.

**Response:** All references are complete and correct, and I have not cited any retracted paper

**Comment 3:** If you would like to make changes to your financial disclosure, please include your updated statement in your cover letter. Guidelines for resubmitting your figure files are available below the reviewer comments at the end of this letter

**Response**: No changes

**Comment 4.** If applicable, we recommend that you deposit your laboratory protocols in [protocols.io](http://protocols.io/) to enhance the reproducibility of your results. Protocols.io assigns your protocol its own identifier (DOI) so that it can be cited independently in the future.

**Response:** Not applicable

**Comment 5:** s the corresponding author, your ORCID iD is verified in the submission system and will appear in the published article. PLOS supports the use of ORCID, and we encourage all coauthors to register for an ORCID iD and use it as well. Please encourage your coauthors to verify their ORCID iD within the submission system before final acceptance, as unverified ORCID iDs will not appear in the published article. Only the individual author can complete the verification step; PLOS staff cannot verify ORCID iDs on behalf of authors.

**Response:** All co-authors have been reminded and encouraged to review their ORCID

Comment 6:   We note that your Data Availability Statement is currently as follows: [All relevant data are within the manuscript.
Please confirm at this time whether or not your submission contains all the raw data required to replicate the results of your study. Authors must share the “minimal data set” for their submission. PLOS defines the minimal data set to consist of the data required to replicate all study findings reported in the article, as well as related metadata and methods (<https://journals.plos.org/plosone/s/data-availability#loc-minimal-data-set-definition>).

**Response:** I confirm that my submission contains all the raw data required to replicate the results of our study without any restrictions

**RIVIEWER 1 COMMENTS**

**Dear Editor**

Thank you for the opportunity to review the manuscript titled *Health care Providers' Perceptions on Barriers to the Provision of Psychosocial Cancer Care at Ocean Road Cancer Institute in Dar es Salaam, Tanzania (PONE-D-25-62143R1).* The authors have sufficiently responded to my previous review. However, I have some minor suggestions/comments for improvement

**COMMENTS**

**Abstract**

- Consider revising the sentence on Line 22 to read: **“*This study adopted an exploratory qualitative design.”***

***Response:*** Thank you. The sentence has been revised and now reads **“*This study adopted an exploratory qualitative design.”*** *As per the suggestion (22)*

**Introduction**

***Background***

- To improve the flow of the background section, consider beginning with the paragraph describing the burden of cancer (currently Paragraph 2) and then linking it to the importance of psychosocial care.

**Response:**

- Paragraph 2 has been shifted to the beginning and restructured to include the burden of cancer and linked with the importance of psychosocial care as per the suggestion (45-52)
- To improve clarity, replace ***“This approach facilitates the development of…”*** *with* ***“This understanding will facilitate the development of…”****.*
- **Response:** The sentence that reads ***“****This approach facilitates the development of****…”*** ***has been replaced with*** *‘‘This understanding will facilitate the development of****…” to improve the clarity****.* ( 93-94)

**Methods**

***Study Design***

- Please apply the same wording suggested for the abstract: ***“This study adopted an exploratory qualitative design.”***

**Response***:* The same wording as suggested in the abstract has been used in this section and now reads ***“This study adopted an exploratory qualitative design.”( 99)***

***Data Collection Methods and Procedures***

- In addition to including the interview guide as a supplementary file, it would be helpful to provide a brief summary of the main interview questions used for data collection (Line 148).

**Response**: A summary of the main questions has been added to the section ( 151-156)

***Reflexivity and Trustworthiness***

- This section currently combines reflexivity and trustworthiness, which are distinct concepts in qualitative research.
  - **Reflexivity** refers to the researchers’ critical reflection on how their backgrounds, assumptions, beliefs, and relationships with participants may have influenced the research process and findings.
  - **Trustworthiness** refers to the overall rigour and quality of the study.
- Consider separating these into two distinct sections.
- . **Response:** This is a good observation; I concur with the suggestion, and now these two sections have been separated into two distinct sections with their respective description ( 205-217)

**Results**

- Although Table 2 presents the themes and subthemes, it would be more informative to include examples of related codes.

**Response:** Representative codes have been added to Table 2 to make it more informative (261-263)

- The title of Theme 3 could be refined for smoother wording. Instead of **“Failure to establish…”**, consider:
  - **“Inability to establish…”**, or
  - **“Challenges in establishing...”**

**Response**: Theme 3 has been renamed and now reads *‘‘Challenges in establishing a patient-provider trusting relationship’’ (262 and 348)*

- Ensure that the wording of Theme 1 in the text (Lines 251–252) is consistent with its presentation in Table 2.

**Response**: The wording of theme 1 in the text is now consistent with its presentations in Table 2( 262 and 348)

**Conclusion**

- The conclusion is somewhat lengthy. Consider summarizing the key findings and recommendations more concisely, and moving the more detailed recommendations to a separate section titled ***“Implications of the Study.”***
- **Response*:*** The conclusion section has been shortened to include key findings and recommendations, and a new section title, Implications of the study, has been added, in which details of the recommendations have been included (484-510 )

**Academic editor comments**

| **s/n** | **Editor comment** | **Authors Response** |
| --- | --- | --- |
| 1. | If the reviewer comments include a recommendation to cite specific previously published works, please review and evaluate these publications to determine whether they are relevant and should be cited. There is no requirement to cite these works unless the editor has indicated otherwise | No recommendation was raised by reviewers who requested to cite previously published work |
|  | Please review your reference list to ensure that it is complete and correct. If you have cited papers that have been retracted, please include the rationale for doing so in the manuscript text, or remove these references and replace them with relevant current references. Any changes to the reference list should be mentioned in the rebuttal letter that accompanies your revised manuscript. If you need to cite a retracted article, indicate the article’s retracted status in the References list and also include a citation and full reference for the retraction notice. | All references are complete and correct, and I have not cited any retracted paper |
| 3 | If you would like to make changes to your financial disclosure, please include your updated statement in your cover letter. Guidelines for resubmitting your figure files are available below the reviewer comments at the end of this letter | No changes |
| 4 | If applicable, we recommend that you deposit your laboratory protocols in [protocols.io](http://protocols.io/) to enhance the reproducibility of your results. Protocols.io assigns your protocol its own identifier (DOI) so that it can be cited independently in the future. | Not applicable |
| 5 | The corresponding author, your ORCID iD is verified in the submission system and will appear in the published article. PLOS supports the use of ORCID, and we encourage all coauthors to register for an ORCID iD and use it as well. Please encourage your coauthors to verify their ORCID iD within the submission system before final acceptance, as unverified ORCID iDs will not appear in the published article. Only the individual author can complete the verification step; PLOS staff cannot verify ORCID iDs on behalf of authors. | All co-authors have been reminded and encouraged to review their ORCID |
| 6 | Comment 6:   We note that your Data Availability Statement is currently as follows: [All relevant data are within the manuscript. Please confirm at this time whether or not your submission contains all the raw data required to replicate the results of your study. Authors must share the “minimal data set” for their submission. PLOS defines the minimal data set to consist of the data required to replicate all study findings reported in the article, as well as related metadata and methods (<https://journals.plos.org/plosone/s/data-availability#loc-minimal-data-set-definition>). | I confirm that my submission contains all the raw data required to replicate the results of our study without any restrictions |

**RESPONSES TO REVIEWER 1 COMMENTS**

| **S/N** | **Manuscript Section** | **Reviewer comments** | **Authors responses** | **Page/line number** |
| --- | --- | --- | --- | --- |
| 1. | Abstract | - Consider revising the sentence on Line 22 to read: **“*This study adopted an exploratory qualitative design.”*** | Thank you. The sentence has been revised and now reads **“*This study adopted an exploratory qualitative design.”*** *As per the suggestion* | 22 |
| 2. | Introduction  (a)Backgrounds | - To improve the flow of the background section, consider beginning with the paragraph describing the burden of cancer (currently Paragraph 2) and then linking it to the importance of psychosocial care. - To improve clarity, replace ***“This approach facilitates the development of…”*** *with* ***“This understanding will facilitate the development of…”****.* | - Paragraph 2 has been shifted to the beginning and restructured to include the burden of cancer and linked with the importance of psychosocial care as per the suggestion - The sentence that reads ***“****This approach facilitates the development of****…”*** *with* ***“has been replaced with*** *‘‘This understanding will facilitate the development of****…” to improve the clarity****.* | 45-53  93-94 |
|  | **Methods** | ***Study Design***   - Please apply the same wording suggested for the abstract: ***“This study adopted an exploratory qualitative design.”***   ***Data Collection Methods and Procedures***   - In addition to including the interview guide as a supplementary file, it would be helpful to provide a brief summary of the main interview questions used for data collection (Line 148).   ***Reflexivity and Trustworthiness***   - This section currently combines reflexivity and trustworthiness, which are distinct concepts in qualitative research.   - **Reflexivity** refers to the researchers’ critical reflection on how their backgrounds, assumptions, beliefs, and relationships with participants may have influenced the research process and findings.   - **Trustworthiness** refers to the overall rigour and quality of the study. - Consider separating these into two distinct sections. | The same wording as suggested in the abstract has been used in this section and now reads ***“This study adopted an exploratory qualitative design.”***  A summary of the main questions has been added to the section  This is good observation; I concur with suggestion and now these two sections have been separated into two distinct sections with their respective description | 99  151-156  205-217 |
|  | **Results:** | **Results**   - Although Table 2 presents the themes and subthemes, it would be more informative to include examples of related codes. - The title of Theme 3 could be refined for smoother wording. Instead of **“Failure to establish…”**, consider:   - **“Inability to establish…”**, or   - **“Challenges in establishing...”** - Ensure that the wording of Theme 1 in the text (Lines 251–252) is consistent with its presentation in Table 2. | Representative codes have been added to table 2 for it to be more informative  The theme 3 has been renamed and now reads *‘‘Challenges in establishing a patient-provider trusting relationship’’*  The wording of theme 1 in the text is now consistent with its presentation in Table 2 | 261-263  262-263 and 348  262 - 348 |
|  | **Conclusions** | - The conclusion is somewhat lengthy. Consider summarizing the key findings and recommendations more concisely, and moving the more detailed recommendations to a separate section titled ***“Implications of the Study.”*** | The conclusion section has been shortened to include key findings and recommendations, and a new section title, Implications of the study, has been added, in which details of the recommendations have been included | 484-510 |
