# Supplementary material for: Healthcare providers’ perceptions on barriers to the provision of psychosocial cancer care at Ocean Road Cancer Institute in Dar es Salaam, Tanzania
Source: PLoS One. 2026 Oct 28;21(10):e0361460. doi: 10.1371/journal.pone.0361460 (PMC13716222; doi:10.1371/journal.pone.0361460)
Supplement: Supplementary file 8 [file pone.0361460.s001.docx]

**PONE-D-25-62-143**

**HealthCare Providers’ Perceptions to provision of psychosocial cancer care at Ocean Road Cancer Institute in Dar es Salaam, Tanzania**

**General comments**

The manuscript presents essential findings on how health care providers strive to meet psychosocial needs of their clients in a low-middle income country. The methods are appropriate; however, the writing style should be revised. The findings are well presented per thematic analysis approach. The quotes are well distributed to reflect representatives of the voices from the various participants. To a large extent, the discussion and conclusion are well written.

**Specific comments**

**Abstract**

Line 22-23, Delete ‘on perceived barriers to psychosocial care provision’ because this is a repletion.

Line 25: Replace ‘method’ with ‘guide’

Line 26: Delete ‘using an interview guide’

Line 37: Change ‘establish’ to establishment of’ and ‘develop’ to ‘development of’

**Materials and Methods**

Line 96: Delete ‘study with’ and ‘aiming at barriers on provision of’

Line 97: Delete ‘psychosocial care from health care providers perspectives’ and replace ‘us’ with the ‘authors’

Line 104: Replace ‘the’ with ‘Tanzania’ and change ‘neighbour’ with ‘neighbouring’

Line 112: Revise ‘is are’ to increase clarity.

**Data collection**

Use the Consolidated criteria for reporting qualitative research (COREQ): a 32-item checklist. This checklist will significantly improve your writing style.

Revise the grammatical errors from lines 123 -141. The last line in this section should read ‘The interviews lasted between 30 and 48 minutes’.

Data quality and management and Data analysis process should be revised.

I observed a number of repetitions, contradictions and overlaps between the two sections. Example, in the data quality and management the authors used content analysis contrary to the data analysis where they used thematic analysis. Revise the lines 149 – 152, “Each transcript was then analysed….coping methods developed to live with illness”. It is difficult to connect this information with the presented findings. Please, revise and be consistent.

Line 175: Replace ‘informal meetings with authors…’ with ‘discussions were held between KA and DAM to reach agreements.

**Findings**

Most of the parts from this section will benefit from English/language editing.
