# Supplementary material for: Healthcare providers’ perceptions on barriers to the provision of psychosocial cancer care at Ocean Road Cancer Institute in Dar es Salaam, Tanzania
Source: PLoS One. 2026 Oct 28;21(10):e0361460. doi: 10.1371/journal.pone.0361460 (PMC13716222; doi:10.1371/journal.pone.0361460)
Supplement: Supplementary file 9 [file pone.0361460.s002.docx]

Dear Editor

I am writing to provide my feedback on the manuscript ID PONE-D-25-62143, entitled “Health care providers perception on barriers for provision of psychosocial cancer care at Ocean Road Cancer Institute in Dar es Salaam, Tanzania” The study addresses an important and underexplored topic in cancer care in Tanzania with potential to contribute meaningfully to the literature. The attached comments are provided to guide authors in improving the manuscript for publication consideration.

**General / Language**

The manuscript contains several grammatical errors, and therefore, language editing is necessary

**Abstract**

Line 17: Replace *“quality of live”* with *“quality of life.”*

**Background**

- Terms such as "psychosocial care" and "psychosocial cancer care" are used interchangeably. The authors should use one preferred term consistently.
- In Lines 75–78, you note that the country has made efforts to implement psychosocial care for cancer patients. Please expand this section by describing these efforts in more detail to enrich the background. Specifically, outline existing policies, programs, guidelines, or initiatives in Tanzania, including how they have been applied in cancer care settings. Additionally, enhance the literature review by incorporating more recent and relevant references, particularly studies from Sub-Saharan Africa, to provide a regional context and strengthen the rationale for your study.

**Methods**

- Lines 110–113: Correct typographical errors and revise the sentence for clarity.
- Line 111: Clarify the composition of the palliative care team. Were medical doctors included?
- Describe the study participants clearly, including the number from each professional discipline/cadre.
- Line 118: Clearly describe the predetermined criteria referred to.
- Line 131: The abstract reports the use of in-depth interviews, while the Methods section describes semi-structured interviews. Please clarify this inconsistency.
- Line 147: The manuscript states that qualitative content analysis was used; however, Line 167 refers to thematic analysis. Please clarify which analytic approach was used and ensure consistency with the abstract.
- Two authors are reported to have participated in data analysis. Please clarify how the remaining two authors contributed to the analysis process.
- The sections on data quality management and data analysis appear inconsistent, as though derived from different studies. Please revise to ensure coherence.
- Line 183-184: Describe what confidentiality/anonymity measures were taken during data collection and reporting
- The authors need to describe measures used to enhance the rigor/trustworthiness of the study

**Findings**

- Provide a summarized table of participants’ background characteristics.
- Line 189: Table 4 is referenced, but Tables 1–3 are not presented. Please clarify or correct the table numbering.
- Line 186: The Results section reports four themes, whereas the abstract reports three. Please resolve this inconsistency and clearly distinguish the main theme from the subthemes.
- The findings appear to predominantly reflect nurses’ perspectives, with only one representation from other healthcare professionals. Please ensure more balanced reporting of views.

**Discussion**

- The authors compare their findings with other studies; however, the comparison is superficial, and there is a need for a more critical comparison.
- Based on the findings, clearly articulate the study’s clinical, educational, and/or policy implications.
- Add a limitations section describing the study’s constraints, including the underrepresentation of certain cadres

**References**

- Some references appear incorrectly formatted. Revise the reference list to ensure all citations are accurate, complete, and appropriately formatted
