# Supplementary material for: Healthcare providers’ perceptions on barriers to the provision of psychosocial cancer care at Ocean Road Cancer Institute in Dar es Salaam, Tanzania
Source: PLoS One. 2026 Oct 28;21(10):e0361460. doi: 10.1371/journal.pone.0361460 (PMC13716222; doi:10.1371/journal.pone.0361460)
Supplement: Supplementary file 10 [file pone.0361460.s003.docx]

MANUSCRIPT REVIEW REPORT:

**Tittle:**

Health care providers perception on barriers for provision of psychosocial cancer care at Ocean Road Cancer Institute in Dar es Salaam, Tanzania

**Authors:**

Kibwanda Athumani1, Nazima Dharsee, Masunga K. Iseselo, Rashidi Heri Kiangi, and Dickson Ally Mkoka1.

**Comments to the Author**

- **General Overview:**

The manuscript addresses psychosocial care for cancer patients. The study will contribute to better plans to provide better care to patients with cancer. The document needs to be edited adequately for example *“Studies show that 54 patient with high level of disease burden, a poor prognosis, young adults aged 18 to 36 years, and elderly aged over 65 years are considered to be at high risk[3–6*]. Thus the manuscript would be very informative and easy to read if substantial line, content and grammar editing are done.

- **Specific Comments:**
  - ***Abstract:*** it is well structured introduction, methods, results, discussions and conclusion
  - ***Introduction:*** readers will benefit more if the manuscript needs to know what does provision of psychosocial care to cancer patients mean or entail? Also, what is known about barriers to psychosocial care and what are the benefits of addressing such barriers including barriers established from the perspective of the care providers
  - The literature review in addition discusses integration and lack of integration of psychosocial care. The conceptual framework that guided the research has to be adequately discussed and made clearer. Thou it is supposed to focus on barriers as perceived by the care providers but it seems to be focusing on integration.
  - Furthermore there is a need to align the title, literature review on integration and research questions see the following sentences. *“Attempts for provision of quality psychosocial care have been there for years, including formulation of standards of care, issuing clinical practice guidelines and distress screening tools development. [4,5,8] . However, these measures have been available internationally but their adoption into clinical practice of routine cancer care have not yet explored 74 especially in developing countries [7,8,11,13]”. This does not support barriers from the perspective of care providers.*
  - ***Research question***: The research question is yet to be clearly stated, in addition the scope of coverage criteria including the conceptual framework should be more detailed and focused.
  - ***Methods:***
    - The methodology used was feasible and effective, but more detail is needed for potential replication. For example, there is need to explain how the study subjects were picked if it was convenient, what defined the convenience.

Also data collection tools and their validation need to be adequately described. At the same the processes like data quality and management would be more informative if it reflects alignment from tittle, objectives, methods and to other sections etc.

The following sentence from the quality and management section is an example of the need to align the manuscript contents.

*“Each transcript was then analyzed for identification of text (meaning units) related to causes of pain, self-management of pain at home, perceived effect of the illness in social and economic life and the management and coping methods developed to live with the illness. The meaning units were condensed and were then extracted. Similar codes were sorted to form categories reflecting the manifest content of the text and similar categories were organized into themes reflecting the latent content of the text”.*

- **Results:**
  - Presentation of results would be more informative if it is guided by objectives. Otherwise, it is not clear to which objectives some of the presented results are responding to and the extent it is supported by preceding texts .
- **Discussions**
  - The section summarizes important findings and are discussed in subsequent paragraphs.
- **Conclusions 1**
  - Conclusion is focusing on integration with very minimal on barriers.
  - Enough room would be needed to make conclusions on barriers.

**Final disposal about the manuscript.**

The manuscript could be published once the observed comments have been addressed

Reviewer: Innocent Semali
