# Supplementary material for: Healthcare providers’ perceptions on barriers to the provision of psychosocial cancer care at Ocean Road Cancer Institute in Dar es Salaam, Tanzania
Source: PLoS One. 2026 Oct 28;21(10):e0361460. doi: 10.1371/journal.pone.0361460 (PMC13716222; doi:10.1371/journal.pone.0361460)
Supplement: Supplementary file 11 [file pone.0361460.s004.docx]

***My comments – PLoS One (09-01-2026)***

**Thank you for the opportunity to review this important manuscript titled: "*Health Care Providers’ Perceptions on barriers to provision of psychosocial cancer care at Ocean Road Cancer Institute in Dar es Salaam, Tanzania*”. This study addresses a significant issue facing cancer patients, especially in low-resource settings. Below are my comments.**

1. **Is the manuscript technically sound, and do the data support the conclusions?**

**Yes.** The study is technically sound and employs a qualitative exploratory study design that aligns with the research objectives. The use of in-depth semi-structured interviews with healthcare providers is appropriate for gathering insights on barriers to psychosocial cancer care. Data analysis through thematic analysis is a suitable approach, providing a credible framework for generating themes. The codes, subthemes, and themes were explained well, supported by relevant quotes. The conclusions are logically derived from the findings. A few additional comments are provided below

2. **Has the statistical analysis been performed appropriately and rigorously?**

**Yes.** The analytical approach (thematic analysis) is well justified and appears to be applied rigorously. However, the author should see a few additional comments below for improvement

3. **Have the authors made all data underlying the findings in their manuscript fully available?**

**Yes.** Most of the data has been described in the results section

4. **Is the manuscript presented in an intelligible fashion and written in standard English?**

**Yes.** The manuscript is generally well written and presented in standard English.

**Additional comments by the Manuscript section**

**Introduction**

1. Check the spelling in line 17 for the sentence, “…….improving patients’ quality of live……” It's supposed to be “life” instead of “live”
2. The first paragraph in the Introduction is very long. Ensure that each paragraph focuses on a single concept. I would suggest lines 43-48 as the first paragraph. The sentence starting with “Cancer is a sharply rising NCD……” as the second paragraph. Sentence starting with “ Evidence suggests that….” as the third paragraph, etc.
3. The sentence, “The lack of proper integration of psychosocial care into routine cancer treatment negatively impacts the emotional and social well-being of patients, leading to a poorer quality of life”, is unnecessary repetition because in the first paragraph, you have already explained the importance of psychosocial care in routine cancer treatment. Here is the opposite of it. You can remove it.
4. The paragraph below is wrongly placed in the introduction section because it distorts the flow of the idea from the first idea. Consider modifying it or removing it. It has no connection to the previous paragraph or the one that follows.

“Integrated care refers to the process of merging all necessarily components of care which are 64 needed to deliver comprehensive care in a way that, all who need it can have quick and easy 65 access of such care[7,8] . In 2008 the Institute of medicine (IOM) pointed out the model on 66 how to deliver integrated care including identification of psychosocial health needs[2] . Few 67 studies indicates how best these diverse psychosocial needs can be met and how this model 68 guides integrated psychosocial care services[7,8,12].”

**Study setting**

5. Consider re-writing this sentence, “….a national tertiary cancer referral hospital located.” I think it's an incomplete sentence.
6. Look at this statement*, “The facility has seven departments which are inpatient, outpatient, nuclear medicine, chemotherapy, radiotherapy, screening and prevention and palliative care”*. I don’t think this sentence is correct. Are they truly departments, or what are they called at ORCI?
7. Look at this statement, “Currently, ORCI does not have specialised units that are responsible for the provision of psychosocial care”. Isn't psychosocial care part of palliative care services? If there is a palliative care unit, what does it do?
8. Look at this statement, “The hospital has so called palliative care team that comprised of palliative care nurses, pharmacists and social workers”. You should also add oncologists, as the head of the palliative care unit is an oncologist at ORCI. It is also high time to suggest the inclusion of Radiation Therapists who have palliative care training. If we really mean integrated care, it is high time to include various professionals working with cancer patients.
9. Look at this statement, “This team is are frontline professionals in provision of psychosocial and supportive care to patients with cancer throughout the continuum of care”. Check spellings and grammar

**Recruitment of study participants**

10. Who are the healthcare providers recruited in your study?
11. Summarise participants’ characteristics in this section. I don’t find it in the results section.
12. Patients would have also provided their perspectives on barriers to psychosocial care. This would have also addressed the existing gap.

**Data quality and management**

13. Who did the daily review of interviews conducted by the first author?

**Data analysis process**

14. Did you use qualitative content analysis or thematic analysis? I can see that in data management, you mentioned qualitative content analysis, and in the analysis section, you explained the use of thematic analysis.
15. Did the authors use any qualitative data analysis software? (e.g., NVivo,), Or was coding done manually?
16. Describe how coding was validated (e.g., through inter-coder agreement or peer debriefing). This would strengthen the methodological trustworthiness.
17. Describe Reflexivity in your study

**Results**

18. The description of Table 4 does not accurately reflect the contents of the table. In the table, I see one theme and three subthemes and not as you described. In the abstract section, you described subthemes as themes.
19. Under the subtheme “Unsupportive organization structure and environment for psychosocial care services”, the first two quotes were based on spiritual services. How do they relate to psychosocial care?

**Discussion**

20. Ensure there is a space between one subtheme you are discussing and the next for easy follow-up.

**References**

21. Look at the reference number 1. It should be written the way it's supposed to be. It is not a research article.
