# Supplementary material for: Healthcare providers’ perceptions on barriers to the provision of psychosocial cancer care at Ocean Road Cancer Institute in Dar es Salaam, Tanzania
Source: PLoS One. 2026 Oct 28;21(10):e0361460. doi: 10.1371/journal.pone.0361460 (PMC13716222; doi:10.1371/journal.pone.0361460)
Supplement: Supplementary file 12 [file pone.0361460.s005.docx]

Kibwanda Athumani,

Muhimbili University of Health and Allied Sciences,

P.O BOX 65001,

Dar es Salaam, Tanzania.

[kibwandaa@gmail.com](mailto:kibwandaa@gmail.com)

13^th^ April 2026.

The Editor-in-Chief,

*PLOS One Journal.*

We are pleased to resubmit a revised manuscript titled **“****Health Care Providers’ Perceptions on Barriers to the Provision of Psychosocial Cancer Care at Ocean Road Cancer Institute in Dar es Salaam, Tanzania”** [**PONE-D-25-62143] - [EMID:12bfc415faca2a14**] Athumani, K, Dharsee, N, Iseselo, M.K, Kiangi, R.K, and Mkoka, D.A, for consideration in the PLOS One Journal.

This is an original research article.

We received comments from the academic editor and four reviewers, which were very useful in improving the quality of our paper’s. We managed to address all the raised recommendations successfully

Our paper explored the perceptions of health care providers working at Ocean Road Cancer Institute regarding the barriers to the provision of psychosocial care to cancer patients. This study revealed that the provision of psychosocial cancer care is limited by inadequate competent staff, lack of coordination, and unsupportive organizational structure, as well as difficulty encountered by health care providers in building a trusting patient-provider therapeutic relationship. Training of staff, establishing coordinating units, developing protocols, and integrating competencies into the training curriculum to address competency gaps. We believe these findings are suitable to be published in the PLOS One Journal due to its scope.

We confirm that this manuscript has not been published elsewhere and is not under consideration by another journal.

Attached are the authors' responses to the academic editor and reviewers’ comments

Yours sincerely

 **Monday 13, 08:07:30 EAT 2026**

Kibwanda Athumani

**Academic Editor comments**

| **S/N** | **Editor comment** | **Authors Response** |
| --- | --- | --- |
| **1** | Please ensure that your manuscript meets PLOS ONE's style requirements, including those for file naming. | The manuscript followed PLOS ONE’s style requirements |
| **2** | Thank you for stating the following financial disclosure: “The Muhimbili University of Health and Allied Sciences supported first author (KA) in data collection process. This was a support to help a student in accomplishing master studies in the field of oncology. KA received a total amount of Tsh.1,500,000/=”  Please state what role the funders took in the study. If the funders had no role, please state: "The funders had no role in study design, data collection and analysis, decision to publish, or preparation of the manuscript." If this statement is not correct you must amend it as needed. Please include this amended Role of Funder statement in your cover letter; we will change the online submission form on your behalf | The funders had no role in study design, data collection and analysis, decision to publish, or preparation of the manuscript |
| **3** | We note that your Data Availability Statement is currently as follows: All relevant data are within the manuscript and its Supporting Information files.  Please confirm at this time whether or not your submission contains all raw data required to replicate the results of your study. Authors must share the “minimal data set” for their submission. PLOS defines the minimal data set to consist of the data required to replicate all study findings reported in the article, as well as related metadata and methods (<https://journals.plos.org/plosone/s/data-availability#loc-minimal-data-set-definition>) | The submission contains all raw data required to replicate the results of our study |
| **4** | lease include captions for your Supporting Information files at the end of your manuscript, and update any in-text citations to match accordingly. Please see our Supporting Information guidelines for more information: <http://journals.plos.org/plosone/s/supporting-information>. | Captions for supporting information file has been included at the end of manuscript and up-date to the in-text citation |
| **5** | If the reviewer comments include a recommendation to cite specific previously published works, please review and evaluate these publications to determine whether they are relevant and should be cited. There is no requirement to cite these works unless the editor has indicated otherwise | No recommendation was raised by reviewers that requested to cite previously published work |

**RESPONSES TO REVIEWER 1 COMMENTS**

| **S/N** | **Manuscript Section** | **Reviewer comments** | **Authors responses** | **Page/line number** |
| --- | --- | --- | --- | --- |
| 1. | Abstract | it is a well-structured introduction, methods, results, discussions and conclusion | Thank you | N/A |
| 2. | Introduction | - readers will benefit more if the manuscript needs to know what does provision of psychosocial care to cancer patients mean or entail? Also, what is known about barriers to psychosocial care and what are the benefits of addressing such barriers including barriers established from the perspective of the care providers - The literature review in addition discusses integration and lack of integration of psychosocial care. The conceptual framework that guided the research has to be adequately discussed and made clearer. Thou it is supposed to focus on barriers as perceived by the care providers but it seems to be focusing on integration. - Furthermore, there is a need to align the title, literature review on integration and research questions see the following sentences. *“Attempts for provision of quality psychosocial care have been there for years, including formulation of standards of care, issuing clinical practice guidelines and distress screening tools development. [4,5,8] . However, these measures have been available internationally but their adoption into clinical practice of routine cancer care have not yet explored 74 especially in developing countries [7,8,11,13]”. This does not support barriers from the perspective of care providers* | Within cancer care units, psychosocial care involves  recognizing and addressing the emotional, psychological, spiritual, and social needs of patients and their families throughout the cancer journey, with the aim of enhancing quality of life and well-being, this statement has been added  The section has been revised and now the focus is on barriers instead of integration across the whole section  I agree with the comment, the section has been revised to align the focus with barriers | 49-52, 61-68 and 74-81  47-94 |
|  | ***Research question*** | The research question is yet to be clearly stated, in addition the scope of coverage criteria including the conceptual framework should be more detailed and focused | The research question focuses on exploring healthcare providers' perceptions of barriers to the provision of psychosocial services at Ocean Road Cancer Institute, Tanzania. This has been clearly stated, and the conceptual framework is now detailed and maintains focus on providers' barriers | 70-94 |
|  | **Methods** | -The methodology used was feasible and effective, but more detail is needed for potential replication. For example, there is need to explain how the study subjects were picked if it was convenient, what defined the convenience.  -Also, data collection tools and their validation need to be adequately described. At the same the processes like data quality and management would be more informative if it reflects alignment from tittle, objectives, methods and to other sections etc.  -The following sentence from the quality and management section is an example of the need to align the manuscript contents.  *“Each transcript was then analyzed for identification of text (meaning units) related to causes of pain, self-management of pain at home, perceived effect of the illness in social and economic life and the management and coping methods developed to live with the illness. The meaning units were condensed and were then extracted. Similar codes were sorted to form categories reflecting the manifest content of the text and similar categories were organized into themes reflecting the latent content of the text”.* | This study used purposive sampling technique to pick study subjects and details for how was operationalised has been added  The data collection tool was validated via expert review, literature review, and modification based on issues that were emerging from each interview. Also, the data quality management section has been reviewed to maintain alignment with other sections  Reviewed to align with the title, methods, and this content has been shifted to the data analysis process section | 125-137  148-158 and 169-182  169-182 |
|  | **Results:** | Presentation of results would be more informative if it is guided by objectives. Otherwise, it is not clear to which objectives some of the presented results are responding to and the extent it is supported by preceding texts. | This manuscript is a part of a big study that aimed to explore barriers and facilitators for psychosocial care provision in which, we had two specific objectives 1. Barriers for psychosocial care provision  2. Facilitators for psychosocial care provision  The findings were too many to be presented in a single manuscript so we decided to split into two manuscripts: one for barriers and the other one will come for facilitators | N/A |
|  | **Discussions** | The section summarizes important findings and are discussed in subsequent paragraphs. | Thank you | N/A |
|  | **Conclusions** | -Conclusion is focusing on integration with very minimal on barriers.  -Enough room would be needed to make conclusions on barriers. | The conclusion section has been enriched with barriers | 466-487 |
|  | **General Overview** | The manuscript addresses psychosocial care for cancer patients. The study will contribute to better plans to provide better care to patients with cancer. The document needs to be edited adequately for example *“Studies show that 54 patient with high level of disease burden, a poor prognosis, young adults aged 18 to 36 years, and elderly aged over 65 years are considered to be at high risk[3–6*]. Thus the manuscript would be very informative and easy to read if substantial line, content and grammar editing are done. | - The content and grammar editing have been done throughout the whole document as per the suggestion | Whole document example 57-59 |

**Response to Reviewer 2 comment**

| S/N | Manuscript section | Reviewer comment | Authors Response | Page/line |
| --- | --- | --- | --- | --- |
| 1. | **Abstract** | Line 17: Replace *“quality of live”* with *“quality of life.”* | *quality of live”* has been replaced with *“quality of life as per the suggestion* | 17 |
| 2. | Background | - Terms such as "psychosocial care" and "psychosocial cancer care" are used interchangeably. The authors should use one preferred term consistently. - In Lines 75–78, you note that the country has made efforts to implement psychosocial care for cancer patients. Please expand this section by describing these efforts in more detail to enrich the background. Specifically, outline existing policies, programs, guidelines, or initiatives in Tanzania, including how they have been applied in cancer care settings. Additionally, enhance the literature review by incorporating more recent and relevant references, particularly studies from Sub-Saharan Africa, to provide a regional context and strengthen the rationale for your study. | - The term psychosocial care" has been agreed to be used as preferred term throughout the document - Initiatives like the establishment of oncology nursing training program at MUHAS, palliative care training, policies, and social welfare national strategic plans have been available as measures to improve psychosocial care in Tanzania. This has been added to the introduction section. Also, recent references from sub-Saharan Africa have been added | Whole document, Example 47-52  83-94 |
| 3. | Methods | - Lines 110–113: Correct typographical errors and revise the sentence for clarity. - Line 111: Clarify the composition of the palliative care team. Were medical doctors included? - Describe the study participants clearly, including the number from each professional discipline/cadre. - Line 118: Clearly describe the predetermined criteria referred to. - Line 131: The abstract reports the use of in-depth interviews, while the Methods section describes semi-structured interviews. Please clarify this inconsistency. - Line 147: The manuscript states that qualitative content analysis was used; however, Line 167 refers to thematic analysis. Please clarify which analytic approach was used and ensure consistency with the abstract. - Two authors are reported to have participated in data analysis. Please clarify how the remaining two authors contributed to the analysis process. - The sections on data quality management and data analysis appear inconsistent, as though derived from different studies. Please revise to ensure coherence. - Line 183-184: Describe what confidentiality/anonymity measures were taken during data collection and reporting - The authors need to describe measures used to enhance the rigor/trustworthiness of the study | - Typographical errors have been removed      - Clarification on the composition of the palliative care team has been included - The number of participants from each professional discipline/cadre has been included - These included healthcare providers working at ORCI in the current unit for at least six months, directly involved in the provision of care to cancer patients, willing to provide informed consent, mentally and physiologically well, and able to communicate either in Swahili or English language - In-depth interview has been replaced with semi-structured interview to maintain consistency - This study used thematic analysis. Revision has been done across the whole document - All authors participated in analysis process, in which KA, started the initial process, which later on the agreed codes, subthemes and themes were shared to DAM, ND, MI and RH for further comments and fine tuning. - Data quality management and data analysis section have been revised to make sure they speak the same idea - During data collection and reporting, no names were used; instead, participants were given an identification number. - A section on how we maintained the rigor of the study has been added | 110–113  117-124  126-129  129-137  25-26  184  188-191  170-182 and 184-200  227-229, and 199-200  201-220 |
|  | Findings | - Provide a summarized table of participants’ background characteristics. - Line 189: Table 4 is referenced, but Tables 1–3 are not presented. Please clarify or correct the table numbering. - Line 186: The Results section reports four themes, whereas the abstract reports three. Please resolve this inconsistency and clearly distinguish the main theme from the subthemes. - The findings appear to predominantly reflect nurses’ perspectives, with only one representation from other healthcare professionals. Please ensure more balanced reporting of views. | - Table of participants' background has been added - Table numbering has been corrected where by table 1 for demographic information and table 2 for themes and sub-themes - Thanks for the good observation. We have three themes, and distinctions between the main theme and subtheme have been made, and now there is coherence between the abstract and results reports - Presentation from social workers has been added in some subthemes to maintain balance; however, we had only 3 social workers, which is why sometimes there is little representation of their quotes | 231-239  238 and 241  28-32, and 241-365  303-306,324-326,356-357 |
|  | **Discussion** | - The authors compare their findings with other studies; however, the comparison is superficial, and there is a need for a more critical comparison. - Based on the findings, clearly articulate the study’s clinical, educational, and/or policy implications. - Add a limitations section describing the study’s constraints, including the underrepresentation of certain cadres | - Critical comparison has been added in the discussion of some subthemes - Implications for education, such as integration in training curricula, establishment of units, and in-house training, as well as designing a favorable psychosocial care policy, have been added - The limitation section has been added | 366-464  366-464  489-498 |
|  | **References** | - Some references appear incorrectly formatted. Revise the reference list to ensure all citations are accurate, complete, and appropriately formatted | - Reference list has been revised to ensure accurate and complete citation | 503-611 |

**AUTHOR'S RESPONSE TO REVIEWER # 3 COMMENTS**

| **S/N** | **Manuscript Section** | **Reviewer comments** | **Authors responses** | **Page/line number** |
| --- | --- | --- | --- | --- |
| 1. | Abstract | 1. Check the spelling in line 17 for the sentence, “……. improving patients’ quality of live……” It's supposed to be “life” instead of “live” | 1. The word live has changed to read life as suggested | 17 |
| 2. | Introduction | 2. The first paragraph in the Introduction is very long. Ensure that each paragraph focuses on a single concept. I would suggest lines 43-48 as the first paragraph. The sentence starting with “Cancer is a sharply rising NCD……” as the second paragraph. Sentence starting with “Evidence suggests that….” as the third paragraph, etc.  3. The sentence, “The lack of proper integration of psychosocial care into routine cancer treatment negatively impacts the emotional and social well-being of patients, leading to a poorer quality of life”, is unnecessary repetition because in the first paragraph, you have already explained the importance of psychosocial care in routine cancer treatment. Here is the opposite of it. You can remove it.  4. The paragraph below is wrongly placed in the introduction section because it distorts the flow of the idea from the first idea. Consider modifying it or removing it. It has no connection to the previous paragraph or the one that follows.  “Integrated care refers to the process of merging all necessarily components of care which are 64 needed to deliver comprehensive care in a way that, all who need it can have quick and easy 65 access of such care[7,8] . In 2008 the Institute of medicine (IOM) pointed out the model on 66 how to deliver integrated care including identification of psychosocial health needs[2] . Few 67 studies indicates how best these diverse psychosocial needs can be met and how this model 68 guides integrated psychosocial care services[7,8,12].” | 2.First paragraph has been already shortened as per suggestion. lines 43-48 are now first paragraph. The sentence starting with “Cancer is a sharply rising NC is a second paragraph. Sentence starting with “Evidence suggests that…is now a third paragraph  3. Thanks for Good observation, the sentence has been removed to maintain logical flow of ideas  4. Thanks for such a good observation also. The paragraph has been removed to maintain the good flow of ideas | Lines 47-61  68-75  64-67 |
|  | **Materials and Methods** | **Study setting**  5. Consider re-writing this sentence, “….a national tertiary cancer referral hospital located.” I think it's an incomplete sentence.  6. Look at this statement, “The facility has seven departments which are inpatient, outpatient, nuclear medicine, chemotherapy, radiotherapy, screening and prevention and palliative care”. I don’t think this sentence is correct. Are they truly departments, or what are they called at ORCI?  7. Look at this statement, “Currently, ORCI does not have specialised units that are responsible for the provision of psychosocial care”. Isn't psychosocial care part of palliative care services? If there is a palliative care unit, what does it do?  8. Look at this statement, “The hospital has so called palliative care team that comprised of palliative care nurses, pharmacists and social workers”. You should also add oncologists, as the head of the palliative care unit is an oncologist at ORCI. It is also high time to suggest the inclusion of Radiation Therapists who have palliative care training. If we really mean integrated care, it is high time to include various professionals working with cancer patients.  9. Look at this statement, “This team is our frontline professionals in the provision of psychosocial and supportive care to patients with cancer throughout the continuum of care”. Check spellings and grammar  **Recruitment of study participants**   1. Who are the healthcare providers recruited in your study? 2. Summaries participants’ characteristics in this section. I don’t find it in the results section. 3. Patients would have also provided their perspectives on barriers to psychosocial care. This would have also addressed the existing gap.   **Data quality and management**   4. Who did the daily review of interviews conducted by the first author?   **Data analysis process**   5. Did you use qualitative content analysis or thematic analysis? I can see that in data management, you mentioned qualitative content analysis, and in the analysis section, you explained the use of thematic analysis. 6. Did the authors use any qualitative data analysis software? (e.g., NVivo,), Or was coding done manually? 7. Describe how coding was validated (e.g., through inter-coder agreement or peer debriefing). This would strengthen the methodological trustworthiness. 8. Describe Reflexivity in your study | 5.sentence has been completed  6.Thanks for good observation, these are called sections at ORCI and changes has been done to the statement with further addition elaboration  7.It’s true that psychosocial care is a part of palliative care, however, international guidelines and standards recommend Psychosocial care to stand as a separate unit to prevent delay in care, creation of stigma, missing of majority and contradicting the guidelines. Furthermore, at the facility the team primarily focuses on patients at the end stages of their life, leaving majority of patients without adequate psychosocial care support.  8. During the time of data collection, oncologists were hardly available because of their tight work schedule. I also agree that the radiation therapist should be included in the future study so that we can capture their perspectives also.  9.spellings and grammar has been already corrected  22. We recruited only nurses and social workers; this has been added to the section  23. Summary has been added to be result section  24. Thanks for your good observations, because of the time we failed to include patients, this will be considered in future studies, so has been added in limitation section and conclusion section  25. DAM and ND did a daily review, and has been added to the section  26. Thanks for the good observation. The study used thematic analysis, and changes have been made to maintain consistency  27. In this study, we used manual coding  28. The coding was validated by peer review and has been added to the paper  The reflexivity section has been added | 10-105  110-115  N/A  121-124  126-129  231-239  482-483 and 490-493  N/A  174-175  184  187-189  189-190  201-220 |
|  | Results | 9. The description of Table 4 does not accurately reflect the contents of the table. In the table, I see one theme and three subthemes and not as you described. In the abstract section, you described subthemes as themes. 10. Under the subtheme “Unsupportive organization structure and environment for psychosocial care services”, the first two quotes were based on spiritual services. How do they relate to psychosocial care? | The table and description have been revised to maintain consistency and uniformity as suggested. Also, in the abstract, the word subthemes " has been replaced with theme  In cancer units, psychosocial care involves recognizing and addressing the emotional, psychological, **spiritual**, and social needs of patients and their families throughout the cancer journey, aiming to improve quality of life and well-being [2].Spiritual care is also one of the key components of psychosocial care. | 28, and 241-243  47-52 |
|  | Discussion | 11. Ensure there is a space between one subtheme you are discussing and the next for easy follow-up. | The space issue has been corrected | 366-464 |
|  | References | 12. Look at the reference number 1. It should be written the way it's supposed to be. It is not a research article. | Editing has been done across the whole reference list | 503 |

**RESPONSES TO REVIEWER 4 COMMENTS**

| **S/N** | **Manuscript Section** | **Reviewer comments** | **Authors responses** | **Page/line number** |
| --- | --- | --- | --- | --- |
| 1. | Abstract | - Line 22-23, Delete ‘on perceived barriers to psychosocial care provision’ because this is a repletion. - Line 25: Replace ‘method’ with ‘guide’ - Line 26: Delete ‘using an interview guide’ - Line 37: Change ‘establish’ to establishment of’ and ‘develop’ to ‘development of’ | - on perceived barriers to psychosocial care provision has been deleted - ‘method’ has been replaced with ‘guide’ - ‘Using an interview guide’ deleted - Changes to establishment of and development of has been done | - 22-23, - 25 - Line 26: - Line 38 |
| 2. | Materials and Methods | - Line 96: Delete ‘study with’ and ‘aiming at barriers on provision of’ - Line 97: Delete ‘psychosocial care from health care providers perspectives’ and replace ‘us’ with the ‘authors’ - Line 104: Replace ‘the’ with ‘Tanzania’ and change ‘neighbour’ with ‘neighbouring’ - Line 112: Revise ‘is are’ to increase clarity. | - Deleted ‘ - Deleted and ‘us’ has been replaced with the ‘authors’ - Replaced and ‘neighbour changed to ‘neighbouring.’ - The line has been revised | - Line 97 - Line 98 - Lines 104-105 - Line 113 |
|  | Data collection | - Use the Consolidated criteria for reporting qualitative research (COREQ): a 32-item checklist. This checklist will significantly improve your writing style. - Revise the grammatical errors from lines 123 -141. The last line in this section should read ‘The interviews lasted between 30 and 48 minutes. - Data quality and management and Data analysis process should be revised.I observed a number of repetitions, contradictions and overlaps between the two sections. Example, in the data quality and management the authors used content analysis contrary to the data analysis where they used thematic analysis. - Revise the lines 149 – 152, “Each transcript was then analysed….coping methods developed to live with illness”. It is difficult to connect this information with the presented findings. Please, revise and be consistent. - Line 175: Replace ‘informal meetings with authors…’ with ‘discussions were held between KA and DAM to reach agreements | Used (COREQ) to correct the section  Grammatical errors have been omitted  Data quality and management and data analysis sections have been reviewed to remove contradicting and repetitive information  Revision has been made and all non-aligned information has been omitted  ‘Informal meetings with authors…’ has been replaced with ‘discussions were held between KA and DAM to reach agreements | 140-168  123-168  170-200  149-152  175 |
|  | Findings | Most of the parts from this section will benefit from English/language editing | English and language editing have been done to the whole section | 241-364 |
