# Supplementary material for: Healthcare providers’ perceptions on barriers to the provision of psychosocial cancer care at Ocean Road Cancer Institute in Dar es Salaam, Tanzania
Source: PLoS One. 2026 Oct 28;21(10):e0361460. doi: 10.1371/journal.pone.0361460 (PMC13716222; doi:10.1371/journal.pone.0361460)
Supplement: Supplementary file 13 [file pone.0361460.s006.docx]

**Dear Editor**

Thank you for the opportunity to review the manuscript titled *Health care Providers' Perceptions on Barriers to the Provision of Psychosocial Cancer Care at Ocean Road Cancer Institute in Dar es Salaam, Tanzania (PONE-D-25-62143R1).* The authors have sufficiently responded to my previous review. However, I have some minor suggestions/comments for improvement

**COMMENTS**

**Abstract**

- Consider revising the sentence on Line 22 to read: **“*This study adopted an exploratory qualitative design.”***

**Introduction**

***Background***

- To improve the flow of the background section, consider beginning with the paragraph describing the burden of cancer (currently Paragraph 2) and then linking it to the importance of psychosocial care.
- To improve clarity, replace ***“This approach facilitates the development of…”*** *with* ***“This understanding will facilitate the development of…”****.*

**Methods**

***Study Design***

- Please apply the same wording suggested for the abstract: ***“This study adopted an exploratory qualitative design.”***

***Data Collection Methods and Procedures***

- In addition to including the interview guide as a supplementary file, it would be helpful to provide a brief summary of the main interview questions used for data collection (Line 148).

***Reflexivity and Trustworthiness***

- This section currently combines reflexivity and trustworthiness, which are distinct concepts in qualitative research.
  - **Reflexivity** refers to the researchers’ critical reflection on how their backgrounds, assumptions, beliefs, and relationships with participants may have influenced the research process and findings.
  - **Trustworthiness** refers to the overall rigour and quality of the study.
- Consider separating these into two distinct sections.

**Results**

- Although Table 2 presents the themes and subthemes, it would be more informative to include examples of related codes.
- The title of Theme 3 could be refined for smoother wording. Instead of **“Failure to establish…”**, consider:
  - **“Inability to establish…”**, or
  - **“Challenges in establishing...”**
- Ensure that the wording of Theme 1 in the text (Lines 251–252) is consistent with its presentation in Table 2.

**Conclusion**

- The conclusion is somewhat lengthy. Consider summarizing the key findings and recommendations more concisely, and moving the more detailed recommendations to a separate section titled ***“Implications of the Study.”***
