# Supplementary material for: Healthcare providers’ perceptions on barriers to the provision of psychosocial cancer care at Ocean Road Cancer Institute in Dar es Salaam, Tanzania
Source: PLoS One. 2026 Oct 28;21(10):e0361460. doi: 10.1371/journal.pone.0361460 (PMC13716222; doi:10.1371/journal.pone.0361460)
Supplement: Supplementary file 14 [file pone.0361460.s008.docx]

**Manuscript Title**: Healthcare providers' perceptions of barriers to the provision of psychosocial care at Ocean Road Cancer Institute in Dar es Salaam, Tanzania.

Response to academic editor comments

Thank you to the academic editor for constructive comments. We have considered all comments and revised the whole manuscript to incorporate them. See the matrix of responses below for a detailed look.

**Comment 1.** Although the manuscript is generally clear, a few sections would benefit from minor language polishing to ensure consistency and academic tone. For example, some sentences remain lengthy and could be tightened for readability. Occasional typographical errors persist (e.g., “SeSo” in the revised manuscript). Please ensure you proofread the manuscript thoroughly.

**Response:** Thank you for the good observation. The entire manuscript has been proofread and edited; typing and grammatical errors have been corrected; some long sentences have been shortened where appropriate to improve readability. We have also standardized some terminology across the whole document to maintain consistency

**Comment 2:** The manuscript notes that social workers belong to the nursing section and have unclear roles. It may be helpful to briefly explain how this organizational arrangement affects psychosocial care delivery.

**Response:** Thank you for this helpful observation. It was noted that the organizational placement of social workers brings unclear reporting, referral pathways, fragmentation of services and challenges in multidisciplinary involvement. So, this explanation has been added to the settings, findings, and discussion sections to clarify the influence of this administrative positioning of social workers in the delivery of psychosocial care services.

**Comment 3:** The discussion is strong but could more explicitly connect certain participant quotations to the broader literature. For example, the quote “Sometimes

might have a desire to convey love, empathy… but because of stresses… I find myself unable to do so” (Participant 4) could be more directly linked to burnout literature.

**Response:** We appreciate this observation. The paragraph discussing providers' emotional challenges, stress, fatigue, and burnout has been expanded in the discussion section and linked more explicitly to relevant current evidence regarding psychological impacts and burnout in healthcare providers caring for cancer patients. Other paragraphs have also been revised to align with this comment.

**Comment 4:** The ethics section is complete, but consider adding a brief statement on how confidentiality was maintained during transcription and data storage

**Response:** Thanks for this comment. The explanation of how confidentiality was maintained is available in the data quality and management section. We agreed that this explanation should be reflected under the ethics section, as per the suggestion. Therefore, the brief statement that indicates how confidentiality was maintained during transcription, data storage, transfer, and reporting has been added to this section

**Comment 5:** Ensure to adhere to a reporting guideline such as COREQ.

**Response**: The whole manuscript, especially the methodology section, was revised against the 32-item COREQ Checklist. All relevant information that needed changes was added to strengthen the manuscript

**Manuscript Title**: Healthcare providers perceptions on Barriers to the provision of psychosocial care at Ocean Road Cancer Institute in Dar es Salaam, Tanzania.

**Response to academic editor comments**

Thanks, the academic editor for constructive comments. We have considered all comments and revised the whole manuscript to incorporate them. See the matrix of response below for detailed look.

| **s/no** | **Academic editor comment** | **Authors Response** |
| --- | --- | --- |
| 1 | Although the manuscript is generally clear, a few sections would benefit from minor language polishing to ensure consistency and academic tone. For example, some sentences remain lengthy and could be tightened for readability. Occasional typographical errors persist (e.g., “SeSo” in the revised manuscript). Please ensure to proofread the manuscript thoroughly. | Thank you for the good observation. The entire manuscript has been proofread and edited; typing and grammatical errors have been corrected, and some long sentences have been shortened where appropriate to improve readability. We have also standardized some terminology across the whole document to maintain consistency |
| 2 | The manuscript notes that social workers belong to the nursing section and have unclear roles. It may be helpful to briefly explain how this organizational arrangement affects psychosocial care delivery. | Thank you for this helpful observation. It was noted that the organizational placement of social workers brings unclear reporting, referral pathways, fragmentation of services, and challenges in multidisciplinary involvement. So, this explanation has been added to the settings, findings, and discussion sections to clarify the influence of this administrative positioning of social workers in the delivery of psychosocial care services. |
| 3 | \| The discussion is strong but could more explicitly connect certain participant quotations to the broader literature. For example, the quote “Sometimes might have a desire to convey love, empathy… but because of stresses… I find myself unable to do so” (Participant 4) could be more directly linked to burnout literature. | We appreciate this observation. The paragraph discussing providers' emotional challenges, stress, fatigue, and burnout has been expanded in the discussion section and linked more explicitly to relevant current evidence regarding psychological impacts and burnout in healthcare providers caring for cancer patients. Other paragraphs have also been revised to align with this comment. |
| 4 | The ethics section is complete, but consider adding a brief statement on how confidentiality was maintained during transcription and data storage | Thanks for this comment. Although the explanation of how confidentiality was maintained is available in the data quality and management section. We agreed that this explanation should be reflected under the ethics section as per the suggestion. Therefore, the brief statement that indicates how confidentiality was maintained during transcription, data storage, transfer, and reporting has been added to this section |
| 5 | Ensure to adhere to a reporting guideline such as COREQ. | The whole manuscript, especially the methodology section, was revised against 32 items of the COREQ Checklist. All relevant information that needed changes was added to strengthen the manuscript. |
