# Supplementary material for: Healthcare providers’ perceptions on barriers to the provision of psychosocial cancer care at Ocean Road Cancer Institute in Dar es Salaam, Tanzania
Source: PLoS One. 2026 Oct 28;21(10):e0361460. doi: 10.1371/journal.pone.0361460 (PMC13716222; doi:10.1371/journal.pone.0361460)
Supplement: Peer review history

# Peer review: Decision Letter 0

**DOI:** 10.1371/journal.pone.0361460.r001

**Authors:** Mohamed Gamal Elsehrawy

14 Jan 2026

Thank you for submitting your manuscript to PLOS ONE. After careful consideration, we feel that it has merit but does not fully meet PLOS ONE’s publication criteria as it currently stands. Therefore, we invite you to submit a revised version of the manuscript that addresses the points raised during the review process.

Please submit your revised manuscript by Feb 28 2026 11:59PM. If you will need more time than this to complete your revisions, please reply to this message or contact the journal office at plosone@plos.org. When you're ready to submit your revision, log on to https://www.editorialmanager.com/pone/ and select the 'Submissions Needing Revision' folder to locate your manuscript file.

- A letter that responds to each point raised by the academic editor and reviewer(s). You should upload this letter as a separate file labeled 'Response to Reviewers'.
- A marked-up copy of your manuscript that highlights changes made to the original version. You should upload this as a separate file labeled 'Revised Manuscript with Track Changes'.
- An unmarked version of your revised paper without tracked changes. You should upload this as a separate file labeled 'Manuscript'.

If you would like to make changes to your financial disclosure, please include your updated statement in your cover letter. Guidelines for resubmitting your figure files are available below the reviewer comments at the end of this letter.

If applicable, we recommend that you deposit your laboratory protocols in protocols.io to enhance the reproducibility of your results. Protocols.io assigns your protocol its own identifier (DOI) so that it can be cited independently in the future. For instructions see: https://journals.plos.org/plosone/s/submission-guidelines#loc-laboratory-protocols. Additionally, PLOS ONE offers an option for publishing peer-reviewed Lab Protocol articles, which describe protocols hosted on protocols.io. Read more information on sharing protocols at https://plos.org/protocols?utm_medium=editorial-email&utm_source=authorletters&utm_campaign=protocols.

We look forward to receiving your revised manuscript.

Kind regards,

Mohamed Gamal Elsehrawy

Academic Editor

PLOS One

Journal Requirements:

When submitting your revision, we need you to address these additional requirements.

1. Please ensure that your manuscript meets PLOS ONE's style requirements, including those for file naming. The PLOS ONE style templates can be found at

https://journals.plos.org/plosone/s/file?id=wjVg/PLOSOne_formatting_sample_main_body.pdf and

https://journals.plos.org/plosone/s/file?id=ba62/PLOSOne_formatting_sample_title_authors_affiliations.pdf

2. Thank you for stating the following financial disclosure:

“The Muhimbili University of Health and Allied Sciences supported first author ( KA) in data collection process. This was a support to help a student in accomplishing master studies in the field of oncology. KA received a total amount of Tsh.1,500,000/=”

Please state what role the funders took in the study. If the funders had no role, please state: "The funders had no role in study design, data collection and analysis, decision to publish, or preparation of the manuscript."

If this statement is not correct you must amend it as needed.

Please include this amended Role of Funder statement in your cover letter; we will change the online submission form on your behalf.

3. We note that your Data Availability Statement is currently as follows: All relevant data are within the manuscript and its Supporting Information files.

Please confirm at this time whether or not your submission contains all raw data required to replicate the results of your study. Authors must share the “minimal data set” for their submission. PLOS defines the minimal data set to consist of the data required to replicate all study findings reported in the article, as well as related metadata and methods (https://journals.plos.org/plosone/s/data-availability#loc-minimal-data-set-definition).

For example, authors should submit the following data:

- The values behind the means, standard deviations and other measures reported;

- The values used to build graphs;

- The points extracted from images for analysis.

Authors do not need to submit their entire data set if only a portion of the data was used in the reported study.

If your submission does not contain these data, please either upload them as Supporting Information files or deposit them to a stable, public repository and provide us with the relevant URLs, DOIs, or accession numbers. For a list of recommended repositories, please see https://journals.plos.org/plosone/s/recommended-repositories.

If there are ethical or legal restrictions on sharing a de-identified data set, please explain them in detail (e.g., data contain potentially sensitive information, data are owned by a third-party organization, etc.) and who has imposed them (e.g., an ethics committee). Please also provide contact information for a data access committee, ethics committee, or other institutional body to which data requests may be sent. If data are owned by a third party, please indicate how others may request data access.

4. Please include captions for your Supporting Information files at the end of your manuscript, and update any in-text citations to match accordingly. Please see our Supporting Information guidelines for more information: http://journals.plos.org/plosone/s/supporting-information.

5. If the reviewer comments include a recommendation to cite specific previously published works, please review and evaluate these publications to determine whether they are relevant and should be cited. There is no requirement to cite these works unless the editor has indicated otherwise.

[Note: HTML markup is below. Please do not edit.]

Reviewers' comments:

Reviewer's Responses to Questions

**Comments to the Author**

1. Is the manuscript technically sound, and do the data support the conclusions?

Reviewer #1: Yes

Reviewer #2: Yes

Reviewer #3: Yes

**********

2. Has the statistical analysis been performed appropriately and rigorously?

Reviewer #1: N/A

Reviewer #2: N/A

Reviewer #3: Yes

**********

3. Have the authors made all data underlying the findings in their manuscript fully available?

The PLOS Data policy

Reviewer #1: Yes

Reviewer #2: Yes

Reviewer #3: Yes

**********

4. Is the manuscript presented in an intelligible fashion and written in standard English?

Reviewer #1: No

Reviewer #2: No

Reviewer #3: Yes

**********

Reviewer #1: The manuscript presents essential findings on how health care providers strive to meet psychosocial needs of their clients in a low-middle income country. The methods are appropriate; however, the writing style should be revised. The findings are well presented per thematic analysis approach. The quotes are well distributed to reflect representatives of the voices from the various participants. To a large extent, the discussion and conclusion are well written. The write up of the methods' section need some revision particularly on data quality and management as well as analysis.

English language is needed particularly on the findings' presentation

Reviewer #2: General / Language

The manuscript contains several grammatical errors, and therefore, language editing is necessary

Abstract

Line 17: Replace “quality of live” with “quality of life.”

Background

• Terms such as "psychosocial care" and "psychosocial cancer care" are used interchangeably. The authors should use one preferred term consistently.

• In Lines 75–78, you note that the country has made efforts to implement psychosocial care for cancer patients. Please expand this section by describing these efforts in more detail to enrich the background. Specifically, outline existing policies, programs, guidelines, or initiatives in Tanzania, including how they have been applied in cancer care settings. Additionally, enhance the literature review by incorporating more recent and relevant references, particularly studies from Sub-Saharan Africa, to provide a regional context and strengthen the rationale for your study.

Methods

• Lines 110–113: Correct typographical errors and revise the sentence for clarity.

• Line 111: Clarify the composition of the palliative care team. Were medical doctors included?

• Describe the study participants clearly, including the number from each professional discipline/cadre.

• Line 118: Clearly describe the predetermined criteria referred to.

• Line 131: The abstract reports the use of in-depth interviews, while the Methods section describes semi-structured interviews. Please clarify this inconsistency.

• Line 147: The manuscript states that qualitative content analysis was used; however, Line 167 refers to thematic analysis. Please clarify which analytic approach was used and ensure consistency with the abstract.

• Two authors are reported to have participated in data analysis. Please clarify how the remaining two authors contributed to the analysis process.

• The sections on data quality management and data analysis appear inconsistent, as though derived from different studies. Please revise to ensure coherence.

• Line 183-184: Describe what confidentiality/anonymity measures were taken during data collection and reporting

• The authors need to describe measures used to enhance the rigor/trustworthiness of the study

Findings

• Provide a summarized table of participants’ background characteristics.

• Line 189: Table 4 is referenced, but Tables 1–3 are not presented. Please clarify or correct the table numbering.

• Line 186: The Results section reports four themes, whereas the abstract reports three. Please resolve this inconsistency and clearly distinguish the main theme from the subthemes.

• The findings appear to predominantly reflect nurses’ perspectives, with only one representation from other healthcare professionals. Please ensure more balanced reporting of views.

Discussion

• The authors compare their findings with other studies; however, the comparison is superficial, and there is a need for a more critical comparison.

• Based on the findings, clearly articulate the study’s clinical, educational, and/or policy implications.

• Add a limitations section describing the study’s constraints, including the underrepresentation of certain cadres

References

• Some references appear incorrectly formatted. Revise the reference list to ensure all citations are accurate, complete, and appropriately formatted

Reviewer #3: Thank you for the opportunity to review this important manuscript titled: "Health Care Providers’ Perceptions on barriers to provision of psychosocial cancer care at Ocean Road Cancer Institute in Dar es Salaam, Tanzania”. This study addresses a significant issue facing cancer patients, especially in low-resource settings. Below are my comments.

1. Is the manuscript technically sound, and do the data support the conclusions?

Yes. The study is technically sound and employs a qualitative exploratory study design that aligns with the research objectives. The use of in-depth semi-structured interviews with healthcare providers is appropriate for gathering insights on barriers to psychosocial cancer care. Data analysis through thematic analysis is a suitable approach, providing a credible framework for generating themes. The codes, subthemes, and themes were explained well, supported by relevant quotes. The conclusions are logically derived from the findings. A few additional comments are provided below

2. Has the statistical analysis been performed appropriately and rigorously?

Yes. The analytical approach (thematic analysis) is well justified and appears to be applied rigorously. However, the author should see a few additional comments below for improvement

3. Have the authors made all data underlying the findings in their manuscript fully available?

Yes. Most of the data has been described in the results section

4. Is the manuscript presented in an intelligible fashion and written in standard English?

Yes. The manuscript is generally well written and presented in standard English.

Additional comments by the Manuscript section

Introduction

1. Check the spelling in line 17 for the sentence, “…….improving patients’ quality of live……” It's supposed to be “life” instead of “live”

2. The first paragraph in the Introduction is very long. Ensure that each paragraph focuses on a single concept. I would suggest lines 43-48 as the first paragraph. The sentence starting with “Cancer is a sharply rising NCD……” as the second paragraph. Sentence starting with “ Evidence suggests that….” as the third paragraph, etc.

3. The sentence, “The lack of proper integration of psychosocial care into routine cancer treatment negatively impacts the emotional and social well-being of patients, leading to a poorer quality of life”, is unnecessary repetition because in the first paragraph, you have already explained the importance of psychosocial care in routine cancer treatment. Here is the opposite of it. You can remove it.

4. The paragraph below is wrongly placed in the introduction section because it distorts the flow of the idea from the first idea. Consider modifying it or removing it. It has no connection to the previous paragraph or the one that follows.

“Integrated care refers to the process of merging all necessarily components of care which are 64 needed to deliver comprehensive care in a way that, all who need it can have quick and easy 65 access of such care[7,8] . In 2008 the Institute of medicine (IOM) pointed out the model on 66 how to deliver integrated care including identification of psychosocial health needs[2] . Few 67 studies indicates how best these diverse psychosocial needs can be met and how this model 68 guides integrated psychosocial care services[7,8,12].”

Study setting

5. Consider re-writing this sentence, “….a national tertiary cancer referral hospital located.” I think it's an incomplete sentence.

6. Look at this statement, “The facility has seven departments which are inpatient, outpatient, nuclear medicine, chemotherapy, radiotherapy, screening and prevention and palliative care”. I don’t think this sentence is correct. Are they truly departments, or what are they called at ORCI?

7. Look at this statement, “Currently, ORCI does not have specialised units that are responsible for the provision of psychosocial care”. Isn't psychosocial care part of palliative care services? If there is a palliative care unit, what does it do?

8. Look at this statement, “The hospital has so called palliative care team that comprised of palliative care nurses, pharmacists and social workers”. You should also add oncologists, as the head of the palliative care unit is an oncologist at ORCI. It is also high time to suggest the inclusion of Radiation Therapists who have palliative care training. If we really mean integrated care, it is high time to include various professionals working with cancer patients.

9. Look at this statement, “This team is are frontline professionals in provision of psychosocial and supportive care to patients with cancer throughout the continuum of care”. Check spellings and grammar

Recruitment of study participants

10. Who are the healthcare providers recruited in your study?

11. Summarise participants’ characteristics in this section. I don’t find it in the results section.

12. Patients would have also provided their perspectives on barriers to psychosocial care. This would have also addressed the existing gap.

Data quality and management

13. Who did the daily review of interviews conducted by the first author?

Data analysis process

14. Did you use qualitative content analysis or thematic analysis? I can see that in data management, you mentioned qualitative content analysis, and in the analysis section, you explained the use of thematic analysis.

15. Did the authors use any qualitative data analysis software? (e.g., NVivo,), or was coding done manually?.

16. Describe how coding was validated (e.g., through inter-coder agreement or peer debriefing). This would strengthen the methodological trustworthiness.

17. Describe Reflexivity in your study

Results

18. The description of Table 4 does not accurately reflect the contents of the table. In the table, I see one theme and three subthemes and not as you described. In the abstract section, you described subthemes as themes.

19. Under the subtheme “Unsupportive organization structure and environment for psychosocial care services”, the first two quotes were based on spiritual services. How do they relate to psychosocial care?

Discussion

20. Ensure there is a space between one subtheme you are discussing and the next for easy follow-up.

References

21. Look at the reference number 1. It should be written the way it's supposed to be. It is not a research article.

**********

what does this mean?). If published, this will include your full peer review and any attached files.

If you choose “no”, your identity will remain anonymous but your review may still be made public.

**Do you want your identity to be public for this peer review?**  For information about this choice, including consent withdrawal, please see our Privacy Policy

Reviewer #1: No

Reviewer #2: **Yes:**  Menti Ndile

Reviewer #3: **Yes:**  Furahini Yoram

**********

[NOTE: If reviewer comments were submitted as an attachment file, they will be attached to this email and accessible via the submission site. Please log into your account, locate the manuscript record, and check for the action link "View Attachments". If this link does not appear, there are no attachment files.]

To ensure your figures meet our technical requirements, please review our figure guidelines: https://journals.plos.org/plosone/s/figures

You may also use PLOS’s free figure tool, NAAS, to help you prepare publication quality figures: https://journals.plos.org/plosone/s/figures#loc-tools-for-figure-preparation.

NAAS will assess whether your figures meet our technical requirements by comparing each figure against our figure specifications.

Attachment Submitted filename: REVIEWERS COMMENTS.docx

Attachment Submitted filename: Reviewers comment PONE-D-25-62143.docx

Attachment Submitted filename: ORHI- CANCER PSYCHOISOCIAL CARE PROVIDER.docx

Attachment Submitted filename: My comments - Kibwana Manuscript PLOS ONE.docx

# Author comment: Author response to Decision Letter 1

**DOI:** 10.1371/journal.pone.0361460.r002

15 Apr 2026

Response to Academic Editor comments

Comment: 1 Please ensure that your manuscript meets PLOS ONE's style requirements, including those for file naming.

Response: The manuscript followed PLOS ONE’s style requirements

Comment: 2 Thank you for stating the following financial disclosure:

“The Muhimbili University of Health and Allied Sciences supported first author (KA) in data collection process. This was a support to help a student in accomplishing master studies in the field of oncology. KA received a total amount of Tsh.1,500,000/=”. Please state what role the funders took in the study. If the funders had no role, please state: "The funders had no role in study design, data collection and analysis, decision to publish, or preparation of the manuscript. “If this statement is not correct you must amend it as needed. Please include this amended Role of Funder statement in your cover letter; we will change the online submission form on your behalf

Response: The funders had no role in study design, data collection and analysis, decision to publish, or preparation of the manuscript

Comment: 3 We note that your Data Availability Statement is currently as follows: All relevant data are within the manuscript and its Supporting Information files. Please confirm at this time whether or not your submission contains all raw data required to replicate the results of your study. Authors must share the “minimal data set” for their submission. PLOS defines the minimal data set to consist of the data required to replicate all study findings reported in the article, as well as related metadata and methods (https://journals.plos.org/plosone/s/data-availability#loc-minimal-data-set-definition)

Response: The submission contains all raw data required to replicate the results of our study

Comment: 4 lease include captions for your Supporting Information files at the end of your manuscript, and update any in-text citations to match accordingly. Please see our Supporting Information guidelines for more information: http://journals.plos.org/plosone/s/supporting-information.

Response: Captions for supporting information file has been included at the end of manuscript and up-date to the in-text citation

Comment: 5 If the reviewer comments include a recommendation to cite specific previously published works, please review and evaluate these publications to determine whether they are relevant and should be cited. There is no requirement to cite these works unless the editor has indicated otherwise

Response: No recommendation was raised by reviewers who requested to cite previously published work

RESPONSES TO REVIEWER 1 COMMENTS

Comment: 1. Abstract

it is a well-structured introduction, methods, results, discussions and conclusion

Response: Thank you

Comment: 2. Introduction

o readers will benefit more if the manuscript needs to know what does provision of psychosocial care to cancer patients mean or entail? Also, what is known about barriers to psychosocial care and what are the benefits of addressing such barriers including barriers established from the perspective of the care providers

Response

Within cancer care units, psychosocial care involves recognizing and addressing the emotional, psychological, spiritual, and social needs of patients and their families throughout the cancer journey, with the aim of enhancing quality of life and well-being, this statement has been added (lines 49-52, 61-68 and 74-81).

o The literature review in addition discusses integration and lack of integration of psychosocial care. The conceptual framework that guided the research has to be adequately discussed and made clearer. Thou it is supposed to focus on barriers as perceived by the care providers but it seems to be focusing on integration. ( Lines47-94)

Response

The section has been revised and now the focus is on barriers instead of integration across the whole section

• Furthermore, there is a need to align the title, literature review on integration and research questions see the following sentences. “Attempts for provision of quality psychosocial care have been there for years, including formulation of standards of care, issuing clinical practice guidelines and distress screening tools development. [4,5,8] . However, these measures have been available internationally but their adoption into clinical practice of routine cancer care have not yet explored 74 especially in developing countries [7,8,11,13]”. This does not support barriers from the perspective of care providers

Response: I agree with the comment, the section has been revised to align the focus with barriers (line 47-94)

Comment 3: Research question -The research question is yet to be clearly stated, in addition the scope of coverage criteria including the conceptual framework should be more detailed and focused

Response: The research question focuses on exploring healthcare providers' perceptions of barriers to the provision of psychosocial services at Ocean Road Cancer Institute, Tanzania. This has been clearly stated, and the conceptual framework is now detailed and maintains focus on providers' barriers (lines 70-94)

Comment 4: The methodology used was feasible and effective, but more detail is needed for potential replication. For example, there is a need to explain how the study subjects were picked if it was convenient, and what defined the convenience.

Response: This study used a purposive sampling technique to pick study subjects, and details for how it was operationalised have been added ( lines 125-137)

Comment 5: Also, data collection tools and their validation need to be adequately described. At the same the processes like data quality and management would be more informative if it reflects alignment from tittle, objectives, methods and to other sections etc.

Response: The data collection tool was validated via expert review, literature review, and modification based on issues that were emerging from each interview. Also, the data quality management section has been reviewed to maintain alignment with other sections (lines 148-158 and 169-182)

Comment 6: The following sentence from the quality and management section is an example of the need to align the manuscript contents.

“Each transcript was then analyzed for identification of text (meaning units) related to causes of pain, self-management of pain at home, perceived effect of the illness in social and economic life and the management and coping methods developed to live with the illness. The meaning units were condensed and were then extracted. Similar codes were sorted to form categories reflecting the manifest content of the text and similar categories were organized into themes reflecting the latent content of the text”.

Response: Reviewed to align with the title, methods, and this content has been shifted to the data analysis process section( lines 169-182 )

Comment 7: Presentation of results would be more informative if it is guided by objectives. Otherwise, it is not clear to which objectives some of the presented results are responding to and the extent it is supported by preceding texts.

Response: This manuscript is a part of a big study that aimed to explore barriers and facilitators for psychosocial care provision, in which we had two specific objectives

1. Barriers to psychosocial care provision

2. Facilitators for psychosocial care provision

The findings were too many to be presented in a single manuscript, so we decided to split them into two manuscripts: one for barriers and the other for facilitators

Comment 8: Discussions

The section summarizes important findings and is discussed in subsequent paragraphs.

Response: Thank you

Comment 9: Conclusions

-The conclusion focuses on integration with very minimal barriers.

-Enough room would be needed to make conclusions on barriers.

Response: The conclusion section has been enriched with barriers ( lines 466-487 )

Comment 10: General Overview The manuscript addresses psychosocial care for cancer patients. The study will contribute to better plans to provide better care to patients with cancer. The document needs to be edited adequately for example “Studies show that 54 patient with high level of disease burden, a poor prognosis, young adults aged 18 to 36 years, and elderly aged over 65 years are considered to be at high risk[3–6]. Thus the manuscript would be very informative and easy to read if substantial line, content and grammar editing are done.

Response: The content and grammar editing have been done throughout the whole document as per the suggestion (Whole document example 57-59 )

RESPONSE TO REVIEWER 2 COMMENTS

Comment 1: Abstract

Line 17: Replace “quality of live” with “quality of life.”

Response: quality of live” has been replaced with “quality of life as per the suggestion ( LINE 17)

Comment 2: Background

•Terms such as "psychosocial care" and "psychosocial cancer care" are used interchangeably. The authors should use one preferred term consistently.

Response: The term psychosocial care" has been agreed to be used as the preferred term throughout the document (Whole document, Example 47-52)

•In Lines 75–78, you note that the country has made efforts to implement psychosocial care for cancer patients. Please expand this section by describing these efforts in more detail to enrich the background. Specifically, outline existing policies, programs, guidelines, or initiatives in Tanzania, including how they have been applied in cancer care settings. Additionally, enhance the literature review by incorporating more recent and relevant references, particularly studies from Sub-Saharan Africa, to provide a regional context and strengthen the rationale for your study.

Response: Initiatives like the establishment of oncology nursing training program at MUHAS, palliative care training, policies, and social welfare national strategic plans have been available as measures to improve psychosocial care in Tanzania. This has been added to the introduction section. Also, recent references from sub-Saharan Africa have been added (Lines 83-94)

• Comment 3: Methods Lines 110–113: Correct typographical errors and revise the sentence for clarity.

Response: Typographical errors have been removed (lines 110–113)

• Line 111: Clarify the composition of the palliative care team. Were medical doctors included?

Response: Clarification on the composition of the palliative care team has been included (lines 117-124)

• Describe the study participants clearly, including the number from each professional discipline/cadre. Line 118: Clearly describe the predetermined criteria referred to.

Response: - The number of participants from each professional discipline/cadre has been included. These included healthcare providers working at ORCI in the current unit for at least six months, directly involved in the provision of care to cancer patients, willing to provide informed consent, mentally and physiologically well, and able to communicate either in Swahili or English ( lines 126-129)

• Line 131: The abstract reports the use of in-depth interviews, while the Methods section describes semi-structured interviews. Please clarify this inconsistency.

Response: In-depth interview has been replaced with a semi-structured interview to maintain consistency ( line 129-137)

• Line 147: The manuscript states that qualitative content analysis was used; however, Line 167 refers to thematic analysis. Please clarify which analytic approach was used and ensure consistency with the abstract.

Response: -This study used thematic analysis. Revision has been done across the whole document

( line 25-26 )

• Two authors are reported to have participated in data analysis. Please clarify how the remaining two authors contributed to the analysis process.

Response: It was a typing error. All authors participated in the analysis process, in which KA initiated the initial analysis, and later the agreed-upon codes, subthemes, and themes were shared with DAM, ND, MI, and RH for further comments and fine-tuning. (line 184 )

• The sections on data quality management and data analysis appear inconsistent, as though derived from different studies. Please revise to ensure coherence.

Response: The data quality management and data analysis sections have been revised so that they convey the same idea (lines 188-191)

• Line 183-184: Describe what confidentiality/anonymity measures were taken during data collection and reporting

Response: During data collection and reporting, no names were used; instead, participants were given an identification number. (170-182 and 184-200)

• The authors need to describe measures used to enhance the rigor/trustworthiness of the study

Response: A section on how we maintained the rigor of the study has been added (lines 227-229, 199-200, 201-220)

Comment: Findings

• Provide a summarized table of participants’ background characteristics.

• Response: A summarized Table of participants' background and its respective description has been added( lines 331-239)

• Line 189: Table 4 is referenced, but Tables 1–3 are not presented. Please clarify or correct the table numbering.

• Response: Table numbering has been corrected, where table 1 for demographic information and table 2 for themes and sub-themes (line 238-241)

• Line 186: The Results section reports four themes, whereas the abstract reports three. Please resolve this inconsistency and clearly distinguish the main theme from the subthemes.

Response: Thanks for the good observation. We have three themes, and distinctions between the main theme and subtheme have been made, and now there is coherence between the abstract and results reports (lines 28-32, and 241-365 )

• The findings appear to predominantly reflect nurses’ perspectives, with only one representation from other healthcare professionals. Please ensure more balanced reporting of views.

• Response: Presentation from social workers has been added in some subthemes to maintain balance; however, we had only 3 social workers, which is why sometimes there is little representation of their quotes (lines 303-306,324-326,356-357 )

• Comment: Discussion

• The authors compare their findings with other studies; however, the comparison is superficial, and there is a need for a more critical comparison.

• Response: Critical comparison has been added in the discussion of some subthemes ( Lines 366-464)

• Based on the findings, clearly articulate the study’s clinical, educational, and/or policy implications.

• Response: Implications for education, such as integration in training curricula, establishment of units, and in-house training, as well as designing a favorable psychosocial care policy, have been added (Lines 366-464)

• Add a limitations section describing the study’s constraints, including the underrepresentation of certain cadres

• Response: The limitation section has been added (Lines 489-498)

• Comment: References

• Some references appear incorrectly formatted. Revise the reference list to ensure all citations are accurate, complete, and appropriately formatted

• Response: Reference list has been revised to ensure accurate and complete citation (lines 503-611)

AUTHOR'S RESPONSE TO REVIEWER # 3 COMMENTS

Comment: 1. Check the spelling in line 17 for the sentence, “……. improving patients’ quality of live……” It's supposed to be “life” instead of “live”

Response: 1. The word live has changed to read life as suggested ( line 17 )

Comment: 2. The first paragraph in the Introduction is very long. Ensure that each paragraph focuses on a single concept. I would suggest lines 43-48 as the first paragraph. The sentence starting with “Cancer is a sharply rising NCD……” as the second paragraph. Sentence starting with “Evidence suggests that….” as the third paragraph, etc.

Response: First paragraph has already been shortened as per the suggestion. Lines 43-48 are now the first paragraph. The sentence starting with “Cancer is a sharply rising NC is in the second paragraph. Sentence starting with “Evidence suggests that…is now a third paragraph ( lines 47-61 )

3. The sentence, “The lack of proper integration of psychosocial care into routine cancer treatment negatively impacts the emotional and social well-being of patients, leading to a poorer quality of life”, is unnecessary repetition because in the first paragraph, you have already explained the importance of psychosocial care in routine cancer treatment. Here is the opposite of it. You can remove it.

Response: Thanks for the good observation. The sentence has been removed to maintain the logical flow of ideas ( Lines 68-75 )

4.The paragraph below is wrongly placed in the introduction section because it distorts the flow of the idea from the first idea. Consider modifying it or removing it. It has no connection to the previous paragraph or the one that follows.

“Integrated care refers to the process of merging all necessarily components of care which are 64 needed to deliver comprehensive care in a way that, all who need it can have quick and easy 65 access of such care[7,8] . In 2008 the Institute of medicine (IOM) pointed out the model on 66 how to deliver integrated care including identification of psychosocial health needs[2] . Few 67 studies indicates how best these diverse psychosocial needs can be met and how this model 68 guides integrated psychosocial care services[7,8,12].”

Response: Thanks for such a good observation also. The paragraph has been removed to maintain the good flow of ideas (Lines 64-67)

Comment: Materials and Methods

Study setting

5. Consider re-writing this sentence, “….a national tertiary cancer referral hospital located.” I think it's an incomplete sentence.

Response: sentence has been completed (110-105 )

6. Look at this statement, “The facility has seven departments which are inpatient, outpatient, nuclear medicine, chemotherapy, radiotherapy, screening and prevention and palliative care”. I don’t think this sentence is correct. Are they truly departments, or what are they called at ORCI?

Response: Thanks for the good observation. These are called sections at ORCI, and changes have been made to the statement with further addition and elaboration ( lines 110-115 ).

7. Look at this statement, “Currently, ORCI does not have specialised units that are responsible for the provision of psychosocial care”. Isn't psychosocial care part of palliative care services? If there is a palliative care unit, what does it do?

Response: It’s true that psychosocial care is a part of palliative care, however, international guidelines and standards recommend Psychosocial care to stand as a separate unit to prevent delay in care, creation of stigma, missing of majority and contradicting the guidelines. Furthermore, at the facility, the team primarily focuses on patients at the end stages of their life, leaving majority of patients without adequate psychosocial care support.

8. Look at this statement, “The hospital has so called palliative care team that comprised of palliative care nurses, pharmacists and social workers”. You should also add oncologists, as the head of the palliative care unit is an oncologist at ORCI. It is also high time to suggest the inclusion of Radiation Therapists who have palliative care training. If we really mean integrated care, it is high time to include various professionals working with cancer patients.

Response: During the time of data collection, oncologists were hardly available because of their tight work schedule. I also agree that the radiation therapist should be included in the future study so that we can capture their perspectives also. (lines 121-124)

9. Look at this statement, “This team is our frontline professionals in the provision of psychosocial and supportive care to patients with cancer throughout the continuum of care”. Check spellings and grammar

Response: Spellings and grammar have already been corrected (lines 126-129)

Recruitment of study participants

1. Who are the healthcare providers recruited in your study?

Response: We recruited only nurses and social workers; this has been added to the section ( lines 231-239 )

2. Summarize participants’ characteristics in this section. I don’t find it in the results section.

Response: Summary has been added to the result section (Lines 482-483,490-493)

3. Patients would have also provided their perspectives on barriers to psychosocial care. This would have also addressed the existing gap.

Response: Thanks for your good observations. Because of the time we failed to include patients, this will be considered in future studies, so it has been added in the limitation section and the conclusion section (lines N/A)

Data quality and management

1. Who did the daily review of interviews conducted by the first author?

Response: DAM and ND did a daily review, and have been added to the section( Lines 174-175 )

Data analysis process

1. Did you use qualitative content analysis or thematic analysis? I can see that in data management, you mentioned qualitative content analysis, and in the analysis section, you explained the use of thematic analysis.

Response: Thanks for the good observation. The study used thematic analysis, and changes have been made to maintain consistency (Lines 184)

2. Did the authors use any qualitative data analysis software? (e.g., NVivo,), Or was coding done manually?

Response: In this study, we used manual coding (Lines 187-189)

3. Describe how coding was validated (e.g., through inter-coder agreement or peer debriefing). This would strengthen the methodological trustworthiness.

Response: The coding was validated by peer review and has been added to the paper (Lines 189-190

4. Describe Reflexivity in your study

Response: The reflexivity section has been added (Lines 210-220)

Comment: Results

5. The description of Table 4 does not accurately reflect the contents of the table. In the table, I see one theme and three subthemes and not as you described. In the abstract section, you described subthemes as themes.

Response: The table and description have been revised to maintain consistency and uniformity as suggested. Also, in the abstract, the word subthemes " has been replaced with theme ( Lines 28, and 241-243)

6. Under the subtheme “Unsupportive organization structure and environment for psychosocial care services”, the first two quotes were based on spiritual services. How do they relate to psychosocial care?

Response: In cancer units, psychosocial care involves recognizing and addressing the emotional, psychological, spiritual, and social needs of patients and their families throughout the cancer journey, aiming to improve quality of life and well-being [2].Spiritual care is also one of the key components of psychosocial care ( Lines 47-52 )

7. Comment: Discussion- Ensure there is a space between one subtheme you are discussing and the next for easy follow-up.

Response: The space issue has been corrected (Lines 366-464)

8. Comments: References: Look at the reference number 1. It should be written the way it's supposed to be. It is not a research article.

Response: Editing has been done across the whole reference list ( 503 )

RESPONSES TO REVIEWER 4 COMMENTS

Comment 1: Abstracts-Line

1. 22-23, Delete ‘on perceived barriers to psychosocial care provision’ because this is a repetition.

2. Line 25: Replace ‘method’ with ‘guide.’

3. Line 26: Delete ‘using an interview guide.’

4. Line 37: Change ‘establish’ to establishment of’ and ‘develop’ to ‘development of.’

Responses

1. perceived barriers to psychosocial care provision have been deleted (lines 22-23)

2. ‘method’ has been replaced with ‘guide. (Lines 25 )

3. ‘Using an interview guide’ deleted (Line 26)

4. Changes to the establishment and development have been made (line 38)

Comment 2: Materials and Methods

1. Line 96: Delete ‘study with’ and ‘aiming at barriers on provision of’

2. Line 97: Delete ‘psychosocial care from health care providers perspectives’ and replace ‘us’ with the ‘authors’

3. Line 104: Replace ‘the’ with ‘Tanzania’ and change ‘neighbour’ with ‘neighbouring’

4. Line 112: Revise ‘is are’ to increase clarity.

Responses

1. Deleted ‘(lines 97)

2. Deleted and ‘us’ has been replaced with the ‘authors’ (Lines 98)

3. Replaced ‘neighbour changed to ‘neighbouring.’ (104-105)

4. The line has been revised (line 113)

Comment 3: Data collection

1. Use the Consolidated criteria for reporting qualitative research (COREQ): a 32-item checklist. This checklist will significantly improve your writing style.

2. Revise the grammatical errors from lines 123 -141. The last line in this section should read ‘The interviews lasted between 30 and 48 minutes.

3. Data quality and management and Data analysis process should be revised.I observed a number of repetitions, contradictions and overlaps between the two sections. Example, in the data quality and management the authors used content analysis contrary to the data analysis where they used thematic analysis.

4. Revise the lines 149 – 152, “Each transcript was then analysed….coping methods developed to live with illness”. It is difficult to connect this information with the presented findings. Please, revise and be consistent.

5. Line 175: Replace ‘informal meetings with authors…’ with ‘discussions were held between KA and DAM to reach agreements

Responses:

1. Used (COREQ) to correct the section (Lines 140-168)

2. Grammatical errors have been omitted (Lines 123-168)

3. Data quality, management, and data analysis sections have been reviewed to remove contradicting and repetitive information (Lines 170-200)

4. Revision has been made, and all non-aligned information has been omitted (Lines 149-152)

5. ‘Informal meetings with authors…’ has been replaced with ‘discussions were held between KA and DAM to reach agreements (Lines 175)

Comment 4:

1. Most of the parts from this section will benefit from English/language editing

Response

1. English and language editing have been done to the whole section (Lines 241-364)

Attachment Submitted filename: Response to Reviewers..docx

# Peer review: Decision Letter 1

**DOI:** 10.1371/journal.pone.0361460.r003

**Authors:** Mohamed Gamal Elsehrawy

17 May 2026

Dear Dr. Athumani,

Thank you for submitting your manuscript to PLOS ONE. After careful consideration, we feel that it has merit but does not fully meet PLOS ONE’s publication criteria as it currently stands. Therefore, we invite you to submit a revised version of the manuscript that addresses the points raised during the review process.

Please submit your revised manuscript by Jul 01 2026 11:59PM. If you will need more time than this to complete your revisions, please reply to this message or contact the journal office at plosone@plos.org. When you're ready to submit your revision, log on to https://www.editorialmanager.com/pone/ and select the 'Submissions Needing Revision' folder to locate your manuscript file.

- A letter that responds to each point raised by the academic editor and reviewer(s). You should upload this letter as a separate file labeled 'Response to Reviewers'.
- A marked-up copy of your manuscript that highlights changes made to the original version. You should upload this as a separate file labeled 'Revised Manuscript with Track Changes'.
- An unmarked version of your revised paper without tracked changes. You should upload this as a separate file labeled 'Manuscript'.

If you would like to make changes to your financial disclosure, please include your updated statement in your cover letter. Guidelines for resubmitting your figure files are available below the reviewer comments at the end of this letter.

If applicable, we recommend that you deposit your laboratory protocols in protocols.io to enhance the reproducibility of your results. Protocols.io assigns your protocol its own identifier (DOI) so that it can be cited independently in the future. For instructions see: https://journals.plos.org/plosone/s/submission-guidelines#loc-laboratory-protocols. Additionally, PLOS ONE offers an option for publishing peer-reviewed Lab Protocol articles, which describe protocols hosted on protocols.io. Read more information on sharing protocols at https://plos.org/protocols?utm_medium=editorial-email&utm_source=authorletters&utm_campaign=protocols.

As the corresponding author, your ORCID iD is verified in the submission system and will appear in the published article. PLOS supports the use of ORCID, and we encourage all coauthors to register for an ORCID iD and use it as well. Please encourage your coauthors to verify their ORCID iD within the submission system before final acceptance, as unverified ORCID iDs will not appear in the published article. *Only*  the individual author can complete the verification step; PLOS staff *cannot*  verify ORCID iDs on behalf of authors.

We look forward to receiving your revised manuscript.

Kind regards,

Mohamed Gamal Elsehrawy

Academic Editor

PLOS One

Journal Requirements:

If the reviewer comments include a recommendation to cite specific previously published works, please review and evaluate these publications to determine whether they are relevant and should be cited. There is no requirement to cite these works unless the editor has indicated otherwise.

Please review your reference list to ensure that it is complete and correct. If you have cited papers that have been retracted, please include the rationale for doing so in the manuscript text, or remove these references and replace them with relevant current references. Any changes to the reference list should be mentioned in the rebuttal letter that accompanies your revised manuscript. If you need to cite a retracted article, indicate the article’s retracted status in the References list and also include a citation and full reference for the retraction notice.

[Note: HTML markup is below. Please do not edit.]

Reviewers' comments:

Reviewer's Responses to Questions

**Comments to the Author**

Reviewer #2: All comments have been addressed

Reviewer #3: All comments have been addressed

Reviewer #4: All comments have been addressed

**********

2. Is the manuscript technically sound, and do the data support the conclusions?

Reviewer #2: Yes

Reviewer #3: Yes

Reviewer #4: Yes

**********

3. Has the statistical analysis been performed appropriately and rigorously?

Reviewer #2: N/A

Reviewer #3: Yes

Reviewer #4: N/A

**********

4. Have the authors made all data underlying the findings in their manuscript fully available?

The PLOS Data policy

Reviewer #2: Yes

Reviewer #3: Yes

Reviewer #4: Yes

**********

5. Is the manuscript presented in an intelligible fashion and written in standard English?

Reviewer #2: Yes

Reviewer #3: Yes

Reviewer #4: Yes

**********

Reviewer #2: After incorporating previous comments, the manuscript is well improved, Authors need to address minor comments attached

Reviewer #3: Authors addressed all my comments. I have no additional comments

Manuscript technically sound

Statistical analysis has been performed appropriately and rigorously

Data are available

Manuscript was written in standard English

Reviewer #4: the authors actually improved the manuscripts. They did English editing. The authors were able to state their position respectfully and in a scholarly manner.

**********

what does this mean?). If published, this will include your full peer review and any attached files.

If you choose “no”, your identity will remain anonymous but your review may still be made public.

**Do you want your identity to be public for this peer review?**  For information about this choice, including consent withdrawal, please see our Privacy Policy

Reviewer #2: **Yes:**  Menti Ndile

Reviewer #3: No

Reviewer #4: No

**********

[NOTE: If reviewer comments were submitted as an attachment file, they will be attached to this email and accessible via the submission site. Please log into your account, locate the manuscript record, and check for the action link "View Attachments". If this link does not appear, there are no attachment files.]

To ensure your figures meet our technical requirements, please review our figure guidelines: https://journals.plos.org/plosone/s/figures

You may also use PLOS’s free figure tool, NAAS, to help you prepare publication quality figures: https://journals.plos.org/plosone/s/figures#loc-tools-for-figure-preparation.

NAAS will assess whether your figures meet our technical requirements by comparing each figure against our figure specifications.

Attachment Submitted filename: Comments PONE-D-25-62143R1.docx

# Author comment: Author response to Decision Letter 2

**DOI:** 10.1371/journal.pone.0361460.r004

2 Jun 2026

Response to Academic Editor comments

Comment: 1 If the reviewer comments include a recommendation to cite specific previously published works, please review and evaluate these publications to determine whether they are relevant and should be cited. There is no requirement to cite these works unless the editor has indicated otherwise

Response: No recommendation was raised by reviewers who requested to cite previously published work

Comment 2. Please review your reference list to ensure that it is complete and correct. If you have cited papers that have been retracted, please include the rationale for doing so in the manuscript text, or remove these references and replace them with relevant current references. Any changes to the reference list should be mentioned in the rebuttal letter that accompanies your revised manuscript. If you need to cite a retracted article, indicate the article’s retracted status in the References list and also include a citation and full reference for the retraction notice.

Response: All references are complete and correct, and I have not cited any retracted paper

Comment 3: If you would like to make changes to your financial disclosure, please include your updated statement in your cover letter. Guidelines for resubmitting your figure files are available below the reviewer comments at the end of this letter

Response: No changes

Comment 4. If applicable, we recommend that you deposit your laboratory protocols in protocols.io to enhance the reproducibility of your results. Protocols.io assigns your protocol its own identifier (DOI) so that it can be cited independently in the future.

Response: Not applicable

Comment 5: s the corresponding author, your ORCID iD is verified in the submission system and will appear in the published article. PLOS supports the use of ORCID, and we encourage all coauthors to register for an ORCID iD and use it as well. Please encourage your coauthors to verify their ORCID iD within the submission system before final acceptance, as unverified ORCID iDs will not appear in the published article. Only the individual author can complete the verification step; PLOS staff cannot verify ORCID iDs on behalf of authors.

Response: All co-authors have been reminded and encouraged to review their ORCID

Comment 6: We note that your Data Availability Statement is currently as follows: [All relevant data are within the manuscript.

Please confirm at this time whether or not your submission contains all the raw data required to replicate the results of your study. Authors must share the “minimal data set” for their submission. PLOS defines the minimal data set to consist of the data required to replicate all study findings reported in the article, as well as related metadata and methods (https://journals.plos.org/plosone/s/data-availability#loc-minimal-data-set-definition).

Response: I confirm that my submission contains all the raw data required to replicate the results of our study without any restrictions

RIVIEWER 1 COMMENTS

Dear Editor

Thank you for the opportunity to review the manuscript titled Health care Providers' Perceptions on Barriers to the Provision of Psychosocial Cancer Care at Ocean Road Cancer Institute in Dar es Salaam, Tanzania (PONE-D-25-62143R1). The authors have sufficiently responded to my previous review. However, I have some minor suggestions/comments for improvement

COMMENTS

Abstract

• Consider revising the sentence on Line 22 to read: “This study adopted an exploratory qualitative design.”

Response: Thank you. The sentence has been revised and now reads “This study adopted an exploratory qualitative design.” As per the suggestion (22)

Introduction

Background

• To improve the flow of the background section, consider beginning with the paragraph describing the burden of cancer (currently Paragraph 2) and then linking it to the importance of psychosocial care.

Response:

• Paragraph 2 has been shifted to the beginning and restructured to include the burden of cancer and linked with the importance of psychosocial care as per the suggestion (45-52)

• To improve clarity, replace “This approach facilitates the development of…” with “This understanding will facilitate the development of…”.

• Response: The sentence that reads “This approach facilitates the development of…” has been replaced with ‘‘This understanding will facilitate the development of…” to improve the clarity. ( 93-94)

Methods

Study Design

• Please apply the same wording suggested for the abstract: “This study adopted an exploratory qualitative design.”

Response: The same wording as suggested in the abstract has been used in this section and now reads “This study adopted an exploratory qualitative design.”( 99)

Data Collection Methods and Procedures

• In addition to including the interview guide as a supplementary file, it would be helpful to provide a brief summary of the main interview questions used for data collection (Line 148).

Response: A summary of the main questions has been added to the section ( 151-156)

Reflexivity and Trustworthiness

• This section currently combines reflexivity and trustworthiness, which are distinct concepts in qualitative research.

� Reflexivity refers to the researchers’ critical reflection on how their backgrounds, assumptions, beliefs, and relationships with participants may have influenced the research process and findings.

� Trustworthiness refers to the overall rigour and quality of the study.

• Consider separating these into two distinct sections.

• . Response: This is a good observation; I concur with the suggestion, and now these two sections have been separated into two distinct sections with their respective description ( 205-217)

Results

• Although Table 2 presents the themes and subthemes, it would be more informative to include examples of related codes.

Response: Representative codes have been added to Table 2 to make it more informative (261-263)

• The title of Theme 3 could be refined for smoother wording. Instead of “Failure to establish…”, consider:

� “Inability to establish…”, or

� “Challenges in establishing...”

Response: Theme 3 has been renamed and now reads ‘‘Challenges in establishing a patient-provider trusting relationship’’ (262 and 348)

• Ensure that the wording of Theme 1 in the text (Lines 251–252) is consistent with its presentation in Table 2.

Response: The wording of theme 1 in the text is now consistent with its presentations in Table 2( 262 and 348)

Conclusion

• The conclusion is somewhat lengthy. Consider summarizing the key findings and recommendations more concisely, and moving the more detailed recommendations to a separate section titled “Implications of the Study.”

• Response: The conclusion section has been shortened to include key findings and recommendations, and a new section title, Implications of the study, has been added, in which details of the recommendations have been included (484-510 )

Attachment Submitted filename: Response to Reviewers comments,.docx

# Peer review: Decision Letter 2

**DOI:** 10.1371/journal.pone.0361460.r005

**Authors:** JONATHAN BAYUO

11 Sep 2026

Dear Dr. Athumani,

Thank you for submitting your manuscript to PLOS One. After careful consideration, we feel that it has merit but does not fully meet PLOS One’s publication criteria as it currently stands. Therefore, we invite you to submit a revised version of the manuscript that addresses the points raised during the review process.

Please submit your revised manuscript by Oct 26 2026 11:59PM. If you will need more time than this to complete your revisions, please reply to this message or contact the journal office at plosone@plos.org. When you're ready to submit your revision, log on to https://www.editorialmanager.com/pone/ and select the 'Submissions Needing Revision' folder to locate your manuscript file.

- A letter that responds to each point raised by the academic editor and reviewer(s). You should upload this letter as a separate file labeled 'Response to Reviewers'.
- A marked-up copy of your manuscript that highlights changes made to the original version. You should upload this as a separate file labeled 'Revised Manuscript with Track Changes'.
- An unmarked version of your revised paper without tracked changes. You should upload this as a separate file labeled 'Manuscript'.

If you would like to make changes to your financial disclosure, please include your updated statement in your cover letter. Guidelines for resubmitting your figure files are available below the reviewer comments at the end of this letter.

If applicable, we recommend that you deposit your laboratory protocols in protocols.io to enhance the reproducibility of your results. Protocols.io assigns your protocol its own identifier (DOI) so that it can be cited independently in the future. For instructions see: https://journals.plos.org/plosone/s/submission-guidelines#loc-laboratory-protocols. Additionally, PLOS One offers an option for publishing peer-reviewed Lab Protocol articles, which describe protocols hosted on protocols.io. Read more information on sharing protocols at https://plos.org/protocols?utm_medium=editorial-email&utm_source=authorletters&utm_campaign=protocols.

As the corresponding author, your ORCID iD is verified in the submission system and will appear in the published article. PLOS supports the use of ORCID, and we encourage all coauthors to register for an ORCID iD and use it as well. Please encourage your coauthors to verify their ORCID iD within the submission system before final acceptance, as unverified ORCID iDs will not appear in the published article. *Only*  the individual author can complete the verification step; PLOS staff *cannot*  verify ORCID iDs on behalf of authors.

We look forward to receiving your revised manuscript.

Kind regards,

JONATHAN BAYUO, PhD

Academic Editor

PLOS One

**Journal Requirements:**

If the reviewer comments include a recommendation to cite specific previously published works, please review and evaluate these publications to determine whether they are relevant and should be cited. There is no requirement to cite these works unless the editor has indicated otherwise.

**Additional Editor Comments:**

Thanks to the authors for their revised draft. Some additional work is needed. Kindly see the comments below for your consideration:

1. Although the manuscript is generally clear, a few sections would benefit from minor language polishing to ensure consistency and academic tone. For example: some sentences remain lengthy and could be tightened for readability. Occasional typographical errors persist (e.g., “SeSo” in the revised manuscript). Please ensure to proofread the manuscript thoroughly.

2. The manuscript notes that social workers belong to the nursing section and have unclear roles. It may be helpful to briefly explain how this organisational arrangement affects psychosocial care delivery.

3. The discussion is strong but could more explicitly connect certain participant quotations to the broader literature. For example, the quote “Sometimes I might have a desire to convey love, empathy… but because of stresses… I find myself unable to do so” (Participant 4) could be more directly linked to burnout literature.

4. The ethics section is complete, but consider adding a brief statement on how confidentiality was maintained during transcription and data storage.

5. Ensure to adhere to a reporting guideline such as COREQ etc.

[Note: HTML markup is below. Please do not edit.]

Reviewers' comments:

Reviewer's Responses to Questions

**Comments to the Author**

Reviewer #2: All comments have been addressed

Reviewer #3: All comments have been addressed

**********

2. Is the manuscript technically sound, and do the data support the conclusions?

Reviewer #2: Yes

Reviewer #3: Yes

**********

3. Has the statistical analysis been performed appropriately and rigorously?

Reviewer #2: N/A

Reviewer #3: Yes

**********

4. Have the authors made all data underlying the findings in their manuscript fully available?

The PLOS Data policy

Reviewer #2: Yes

Reviewer #3: Yes

**********

5. Is the manuscript presented in an intelligible fashion and written in standard English?

Reviewer #2: Yes

Reviewer #3: Yes

**********

Reviewer #2: I acknowledge that the authors have addressed all previous comments adequately and there are no current comment

Reviewer #3: (No Response)

**********

what does this mean?). If published, this will include your full peer review and any attached files.

If you choose “no”, your identity will remain anonymous but your review may still be made public.

**Do you want your identity to be public for this peer review?**  For information about this choice, including consent withdrawal, please see our Privacy Policy

Reviewer #2: **Yes:**  Menti Ndile

Reviewer #3: No

**********

[NOTE: If reviewer comments were submitted as an attachment file, they will be attached to this email and accessible via the submission site. Please log into your account, locate the manuscript record, and check for the action link "View Attachments". If this link does not appear, there are no attachment files.]

To ensure your figures meet our technical requirements, please review our figure guidelines: https://journals.plos.org/plosone/s/figures

You may also use PLOS’s free figure tool, NAAS, to help you prepare publication quality figures: https://journals.plos.org/plosone/s/figures#loc-tools-for-figure-preparation.

NAAS will assess whether your figures meet our technical requirements by comparing each figure against our figure specifications.

# Author comment: Author response to Decision Letter 3

**DOI:** 10.1371/journal.pone.0361460.r006

7 Oct 2026

Manuscript Title: Healthcare providers' perceptions of barriers to the provision of psychosocial care at Ocean Road Cancer Institute in Dar es Salaam, Tanzania.

Response to academic editor comments

Thank you to the academic editor for constructive comments. We have considered all comments and revised the whole manuscript to incorporate them. See the matrix of responses below for a detailed look.

Comment 1. Although the manuscript is generally clear, a few sections would benefit from minor language polishing to ensure consistency and academic tone. For example, some sentences remain lengthy and could be tightened for readability. Occasional typographical errors persist (e.g., “SeSo” in the revised manuscript). Please ensure you proofread the manuscript thoroughly.

Response: Thank you for the good observation. The entire manuscript has been proofread and edited; typing and grammatical errors have been corrected; some long sentences have been shortened where appropriate to improve readability. We have also standardized some terminology across the whole document to maintain consistency

Comment 2: The manuscript notes that social workers belong to the nursing section and have unclear roles. It may be helpful to briefly explain how this organizational arrangement affects psychosocial care delivery.

Response: Thank you for this helpful observation. It was noted that the organizational placement of social workers brings unclear reporting, referral pathways, fragmentation of services and challenges in multidisciplinary involvement. So, this explanation has been added to the settings, findings, and discussion sections to clarify the influence of this administrative positioning of social workers in the delivery of psychosocial care services.

Comment 3: The discussion is strong but could more explicitly connect certain participant quotations to the broader literature. For example, the quote “Sometimes

might have a desire to convey love, empathy… but because of stresses… I find myself unable to do so” (Participant 4) could be more directly linked to burnout literature.

Response: We appreciate this observation. The paragraph discussing providers' emotional challenges, stress, fatigue, and burnout has been expanded in the discussion section and linked more explicitly to relevant current evidence regarding psychological impacts and burnout in healthcare providers caring for cancer patients. Other paragraphs have also been revised to align with this comment.

Comment 4: The ethics section is complete, but consider adding a brief statement on how confidentiality was maintained during transcription and data storage

Response: Thanks for this comment. The explanation of how confidentiality was maintained is available in the data quality and management section. We agreed that this explanation should be reflected under the ethics section, as per the suggestion. Therefore, the brief statement that indicates how confidentiality was maintained during transcription, data storage, transfer, and reporting has been added to this section

Comment 5: Ensure to adhere to a reporting guideline such as COREQ.

Response: The whole manuscript, especially the methodology section, was revised against the 32-items COREQ Checklist. All relevant information that needed changes was added to strengthen the manuscript

Attachment Submitted filename: Ressponse to Reviewers.docx

# Editor report: Decision Letter 3

**DOI:** 10.1371/journal.pone.0361460.r007

**Authors:** JONATHAN BAYUO

12 Oct 2026

Healthcare Providers' Perceptions on Barriers to the Provision of Psychosocial Cancer Care at Ocean Road Cancer Institute in Dar es Salaam, Tanzania

PONE-D-25-62143R3

Dear Dr. Athumani,

We’re pleased to inform you that your manuscript has been judged scientifically suitable for publication and will be formally accepted for publication once it meets all outstanding technical requirements.

Within one week, you’ll receive an e-mail detailing the required amendments. When these have been addressed, you’ll receive a formal acceptance letter and your manuscript will be scheduled for publication.

An invoice will be generated when your article is formally accepted. Please note, if your institution has a publishing partnership with PLOS and your article meets the relevant criteria, all or part of your publication costs will be covered. Please make sure your user information is up-to-date by logging into Editorial Manager at Editorial Manager® and clicking the ‘Update My Information' link at the top of the page. For questions related to billing, please contact billing support.

If your institution or institutions have a press office, please notify them about your upcoming paper to help maximize its impact. If they’ll be preparing press materials, please inform our press team as soon as possible -- no later than 48 hours after receiving the formal acceptance. Your manuscript will remain under strict press embargo until 2 pm Eastern Time on the date of publication. For more information, please contact onepress@plos.org.

Kind regards,

JONATHAN BAYUO, PhD

Academic Editor

PLOS One

Additional Editor Comments (optional):

Reviewers' comments:

# Editor report: Acceptance letter

**DOI:** 10.1371/journal.pone.0361460.r008

**Authors:** JONATHAN BAYUO

PONE-D-25-62143R3

PLOS One

Dear Dr. Athumani,

I'm pleased to inform you that your manuscript has been deemed suitable for publication in PLOS One. Congratulations! Your manuscript is now being handed over to our production team.

At this stage, our production department will prepare your paper for publication. This includes ensuring the following:

* All references, tables, and figures are properly cited

* All relevant supporting information is included in the manuscript submission,

* There are no issues that prevent the paper from being properly typeset

You will receive further instructions from the production team, including instructions on how to review your proof when it is ready. Please keep in mind that we are working through a large volume of accepted articles, so please give us a few days to review your paper and let you know the next and final steps.

Lastly, if your institution or institutions have a press office, please let them know about your upcoming paper now to help maximize its impact. If they'll be preparing press materials, please inform our press team within the next 48 hours. Your manuscript will remain under strict press embargo until 2 pm Eastern Time on the date of publication. For more information, please contact onepress@plos.org.

You will receive an invoice from PLOS for your publication fee after your manuscript has reached the completed accept phase. If you receive an email requesting payment before acceptance or for any other service, this may be a phishing scheme. Learn how to identify phishing emails and protect your accounts at https://explore.plos.org/phishing.

If we can help with anything else, please email us at customercare@plos.org.

Thank you for submitting your work to PLOS One and supporting open access.

Kind regards,

PLOS One Editorial Office Staff

on behalf of

Dr. JONATHAN BAYUO

Academic Editor

PLOS One
